# Causal effects of the RANK-RANKL-OPG system and scoliosis: A bidirectional 2-sample Mendelian randomization study

**DOI:** 10.1097/MD.0000000000040934

**Published:** 2024-12-13

**Authors:** Wei Xie, Wen-Tao Wan, Shuai-Yi Liu, Jia-Qi Wang, Chao Chen, Xun Sun, Xin-Yu Liu, Qiang Yang

**Affiliations:** a Department of Spine Surgery, Tianjin Hospital, Tianjin University, Tianjin, China; b Tianjin Key Laboratory of Exercise Physiology and Sports Medicine, Institute of Sport, Exercise & Health, Tianjin University of Sport, Tianjin, China; c Graduate School, Tianjin Medical University, Tianjin, China; d The First Clinical Medical College, Liaoning University of Traditional Chinese Medicine, Shenyang, Liaoning, China; e Department of Orthopedics, Qilu Hospital of Shandong University, Shandong University Centre for Orthopedics, Advanced Medical Research Institute, Jinan, Shandong, China.

**Keywords:** bone mass reduction, causal relationship, Mendelian randomization, musculoskeletal diseases, scoliosis

## Abstract

Epidemiological studies and a recent Mendelian randomization (MR) study have identified an association between low bone mass and an increased risk of scoliosis. Previous research suggests that bone loss in patients with scoliosis may be related to the RANK-RANKL-OPG system. This study is to investigate whether a causal relationship exists between the RANK-RANKL-OPG system and the development of scoliosis. Genome-wide association study (GWAS) data for RANK and RANKL were sourced from the UK Biobank’s Pharmaceutical Proteomics Project, while OPG data were derived from 2 independent cohorts, and scoliosis data from the FinnGen R10 database. A bidirectional 2-sample MR framework was applied to investigate causal relationships between OPG, RANK, RANKL, and scoliosis, with inverse variance weighting (IVW) as the main analytical method. Meta-analysis was used to integrate findings across cohorts, and multiple sensitivity analyses were conducted to assess the robustness and reliability of the results. According to the IVW results, there was no significant causal relationship between RANK (OR = 0.973, 95% CI = 0.871–1.087, *P* = .626) and RANKL (OR = 1.048, 95% CI = 0.938–1.171, *P* = .411) and scoliosis. OPG is a potential protective factor for scoliosis (Folkersen 2020 OR = 0.739, 95% CI = 0.611–0.893, *P* = .002; Zhao 2023 OR = 0.833, 95% CI = 0.716–0.968, *P* = .017).The results of Meta-analysis also showed OPG (*P* = 1.428e−4) would reduce the risk of scoliosis. Inverse MR analysis showed no statistically significant causal relationship between scoliosis and RANK, RANKL and OPG levels (*P* > .05). Our study employing MR methodology provides robust evidence supporting a causal relationship between decreased osteoprotegerin (OPG) levels and increased susceptibility to scoliosis. However, no significant relationship was found between scoliosis with the RANK-RANKL-OPG system. This research establishes a basis for further exploration of the pathophysiological mechanisms and potential targeted treatments for scoliosis. Future studies are necessary to understand how OPG influences the development of scoliosis.

## 1. Introduction

Scoliosis is a 3-dimensional spinal deformity characterized by lateral curvature in the coronal plane, altered curvature in the sagittal plane, and vertebral rotation in the transverse plane. The diagnosis of scoliosis is confirmed when the Cobb angle exceeds 10° on standing anteroposterior and posteroanterior total spine radiographs. Although there are multiple etiologies for scoliosis, more than 60% of cases are idiopathic.^[1]^ Severe scoliosis can result in multi-organ complications, including spinal cord compression, pulmonary dysfunction, and cardiovascular diseases. Based on current research, the etiology of scoliosis remains multifactorial, with no singular doctrine or cause providing a comprehensive explanation for the clinicopathological features of the condition. The prevailing consensus among experts suggests that scoliosis arises from a combination of various contributing factors.^[2]^

Bernard first established a connection between bone loss and idiopathic scoliosis in the 1980s.^[3]^ Recent epidemiological studies have demonstrated that many patients with scoliosis display lower bone density and quality in both axial and peripheral skeletal regions, suggesting that diminished bone mass may contribute to the pathogenesis of scoliosis.^[4–6]^ Nuclear factor kB receptor activator (RANK), its ligand (RANKL), and osteoprotegerin (OPG) are key regulators of bone loss. RANKL promotes osteoclast-mediated bone resorption by binding to RANK on osteoblasts and their precursors, while OPG acts as a decoy receptor for RANKL, inhibiting this interaction and thus preventing osteoclast maturation and activation.^[7–9]^ Dysregulation of the RANKL/OPG ratio is a major driver of bone resorption in conditions such as glucocorticoid-induced osteoporosis, chronic inflammatory arthritis, and estrogen deficiency.^[10–12]^ In cases of adolescent idiopathic scoliosis, observed levels of RANK and the RANKL/OPG ratios are inversely related to serum OPG levels and lumbar spine bone mineral density (LSBMD). Serum OPG levels positively correlate with both LSBMD and the BMD in the femoral neck.^[13]^ However, the causal relationship between the RANK-RANKL-OPG system and scoliosis remains inadequately explored.

Mendelian randomization (MR) analysis is a technique used to determine causality. It employs genetic variations as instrumental variables (IVs) to investigate causal relationships with outcomes, thereby bypassing potential confounding factors and the possibility of reverse causation.^[14]^ MR has identified risk factors for various diseases.^[15,16]^ This study employs MR to investigate the causal link between bone loss and scoliosis using data from the GWAS database.

## 2. Materials and methods

### 2.1. Study design

**Figure 1.**
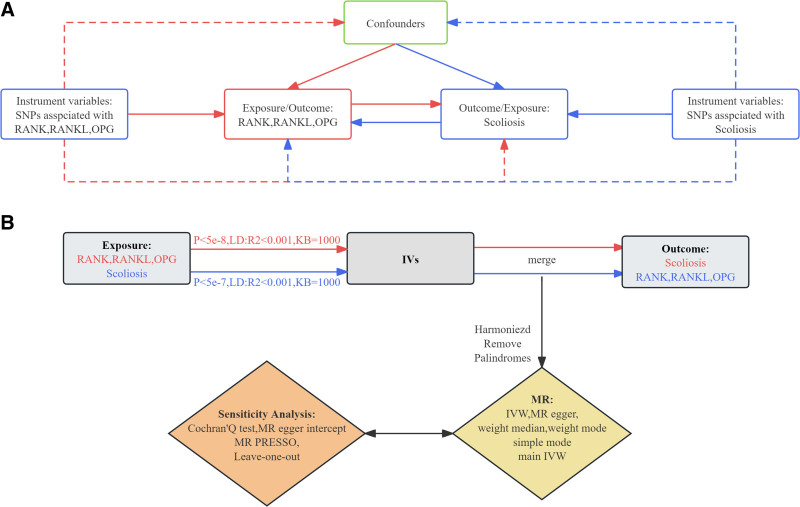
Illustration of the flowchart of the 2-sample bidirectional MR analysis; conceptual diagram of the 3 main hypotheses of the MR analysis. The red line represents the MR analysis of the causal relationship between the RANK-RANKL-OPG system with scoliosis. The blue line represents the MR analysis of the causal relationship between scoliosis with the RANK-RANKL-OPG system. MR = Mendelian randomization, OPG = osteoprotectin, RANK = receptor activator of nuclear factor of kappa-B, RANKL = receptor activator of kappa-B ligand of nuclear factor of kappa-B.

Causal inference using a bidirectional 2-sample MR study (Fig. 1). This MR study was performed based on 3 MR core assumptions (Fig. 1A): There is a strong correlation between IVs and exposure; IVs are independent and unrelated to other confounders; and IVs should not influence outcomes through pathways other than exposure. Figure 1B illustrates the general flowchart of this MR study. Specifically, strongly correlated IVs were screened based on 3 major hypotheses. Subsequently, bidirectional causal relationships between OPG (from 2 distinct cohorts), RANKL, RANK, and scoliosis were analyzed using MR methods. A meta-analysis was then conducted to synthesize the MR results from the 2 cohorts, integrating findings with a random-effects model to account for potential heterogeneity. To conclude, multiple sensitivity analyses were performed to rigorously assess the robustness and reliability of the MR findings.

## 3. Data source

OPG data were derived from GWAS summary statistics of 2 independent cohorts: a recent genome-wide meta-analysis by Folkersen et al, which examined 90 cardiovascular-related proteins and identified 11 with causal links to human disease^[17]^; and a large-scale genome-wide protein quantitative trait loci analysis by Zhao et al, which studied 91 plasma inflammatory proteins and identified multiple genetic variants influencing protein levels.^[18]^ The RANK and RANKL data were both obtained from the Pharmaceutical Proteomics Project of the UK Biobank. Specifically, the RANKL protein is encoded by the gene tumor necrosis factor ligand superfamily member 11 (TNFSF11), while the RANK protein is encoded by the gene tumor necrosis factor receptor superfamily member 11A (TNFRSF11A). GWAS data for RANKL and RANK were retrieved from the UK Biobank by searching using the gene IDs “TNFSF11” for RANKL and “TNFRSF11A” for RANK, respectively.^[19]^ Scoliosis data were obtained from the FinnGen Consortium R10, to ensure population matching with the exposure data and to minimize population stratification bias.^[20]^ All GWAS datasets are listed in Table 1.

**Table 1:** Summary of genetic data information for exposure and outcome phenotypes.

Exposure/outcome	GWAS ID	Sample size	NSNP	People	Year
OPG (Folkersen et al)	ebi-a-GCST90012002	21,758	131,380,39	European	2020
OPG (Zhao et al)	ebi-IPs91-GCST90274830	11,788	NA	European	2023
RANK	ukb-ppp-TNFRSF11A-OID20646	34,049	152,905,20	European	2023
RANKL	ukb-ppp-TNFSF11-OID20592	33,657	152,550,62	European	2023
Scoliosis	finngen_R10_M13_SCOLIOSIS	297,587	203,725,10	European	2023

Abbreviations: OPG = osteoprotectin, RANK = receptor activator of nuclear factor of kappa-B, RANKL = receptor activator of kappa-B ligand of nuclear factor of kappa-B.

## 4. MR analysis principles and selection of instrumental variables

Using genetic variations as IVs, MR can determine causal relationships while controlling for confounding variables and preventing reverse causation. In the context of MR analysis, single nucleotide polymorphisms (SNPs) serve as these IVs. The criteria for selecting IVs, including: SNPs with a strong association with exposure factors (*P* < 5e−8) were chosen; Linkage disequilibrium analysis based on the 1000 Genomes Project European sample data, with parameters set at *r*^2^ < 0.001 and kb = 10,000; Exclusion of palindromic SNPs to ensure proper allelic matching between exposure and outcome; and calculate the *F*-statistic (using the formula: *F* = [*R*^2^ × (*N* − 1 − *K*)]/ [(1 − *R*^2^) × *K*], *N* stands for the sample size of the exposed GWAS data, *K* is the count of SNPs, and *R*^2^ is the variance of the exposure that the SNPs explain), ensuring *F* > 10 to avoid weak instrument bias.^[21]^ The formula for calculating *R*^2^ is *R*^2^ = [2 × β^2^ × EAF × (1 − EAF)]/[2 × β^2^ × EAF × (1 − EAF) + (SE × β^2^) × 2 × *N* × EAF × (1 − EAF)], where EAF is the effect allele frequency, β is the effect size of the allele, and SE is the standard error.^[22]^ In our study, the MR methods used included MR-Egger regression, weighted median (WM), inverse variance weighting (IVW), simple mode, and weighted mode. The IVW method transforms instrumental variable results for exposure effects into weighted regressions, generating an overall estimate of exposure’s impact on outcomes. When horizontal pleiotropy is absent, IVW provides unbiased estimates by minimizing confounding variables’ impact. MR-Egger can be employed when SNPs are pleiotropic, as it is susceptible to influence by peripheral genetic factors, potentially leading to erroneous results. The WM approach offers reliable causal effect estimates by using the majority of genetic variants. Furthermore, even if some IVs do not meet the MR method’s criteria for establishing causality, the WM model remains a viable option. Consequently, we used IVW as the primary analytical method, with additional methods were used as complementary methods for causal analysis.

## 5. Sensitivity analyses

In order to assess the presence of variance, the MR-Egger and IVW methods were tested for heterogeneity using Cochrane *Q* test, which demonstrates significant heterogeneity in the analyses if the test for the Q statistic is significant (*P* < .05).^[23]^ Horizontal polytropy was also tested using MR-polytropy residual sum and outlier (MR-PRESSO) and MR-egger intercept. MR-PRESSO identified and corrected for outliers indicative of pleiotropic bias, while results from global heterogeneity tests with *P*-values >.05 suggested an absence of horizontal pleiotropy.^[24]^ MR-Egger intercept is to assess the relationship of multivalence between IVs and other potential confounders if the result of MR-Egger intercept analysis *P* < .05 indicates the presence of horizontal multivalence.^[25]^ The robustness of the MR analysis results was tested using the leave-one-out (“leave-one-out”) method, which explores whether there is a single SNP driving the causal association by removing individual IVs one by one, and if there is a large effect on the MR analysis after removing a single SNP, it indicates that the MR analysis is affected by a single instrumental variable.

All statistical analyses were performed using *R* version 4.3.3, utilizing the “Two-sample MR” package (version 0.6.1),the “meta “ package (version 7.0-0) and the “MRPRESSO” package. The criterion for significant evidence of a causal effect was established at *P* < .05.

## 6. Results

### 6.1. Causal effects of the RANK-RANKL-OPG system on scoliosis

**Figure 2.**
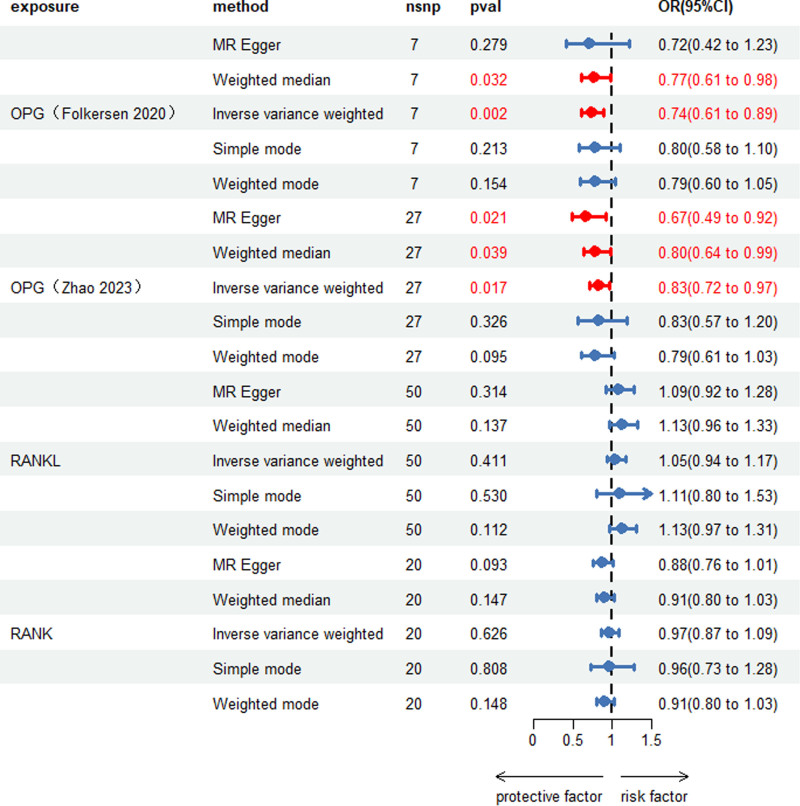
Causal effect of the RANK-RANKL-OPG system on Scoliosis. CI = confidence interval, IVW = inverse variance weighted, NSNP = number of single nucleotide polymorphisms, OPG = osteoprotectin, RANK = receptor activator of nuclear factor of kappa-B, RANKL = receptor activator of kappa-B ligand of nuclear factor of kappa-B.

**Figure 3.**
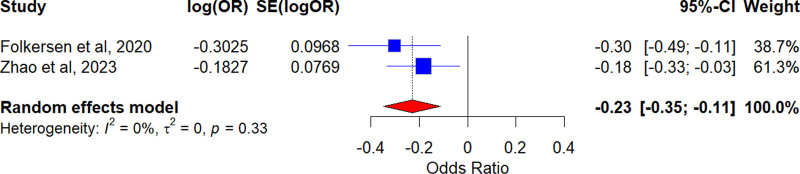
Meta-analysis of IVW results to predict associations of OPG with scoliosis. IVW = inverse variance weighted, OPG = osteoprotectin.

After chain imbalance, deletion of palindromic sequences and weak instrumental variable bias analyses, 7 and 27 IVs were used to assess the causal effect of OPG on scoliosis in the Folkersen et al and Zhao et al cohorts, respectively. A total of 50 SNPs were included in RANKL, and a total of 20 SNPs were included in RANK. In addition, the *F*‐statistics of all IVs were >10. The filtered IVs of interest were analyzed with the outcome data by MR. We mainly used IVW as the method of MR analysis, a meta- analysis of IVW results in both cohorts showed that OPG was a protective factor for scoliosis (Folkersen 2020 – OR = 0.739, 95% CI = 0.611–0.893, *P* = .002; Zhao 2023 – OR = 0.833, 95% CI = 0.716–0.968, *P* = .017); however, no causal relationship was found between RANKL and RANK with scoliosis (RANKL OR = 1.048, 95% CI = 0.938–1.171, *P* = .411; RANK OR = 0.973, 95% CI = 0.871–1.087, *P* = .626) (Fig. 2). The IVW results showed that OPG levels in both cohorts reduced the risk of scoliosis, and to further improve the robustness of the results, we used Meta-analysis to summarize the results of the IVW analysis. The random-effects model results showed a causal relationship between OPG (OR = 0.795, 95% CI = 0.707–0.895, *P* = 1.428e−4) with scoliosis, which were consistent with results of IVW (Fig. 3). Cochrane *Q* test was employed to assess the heterogeneity of the MR-Egger and IVW methods for OPG (Folkersen 2020 IVW: Q_𝑃 =.665, MR-Egger: Q_*P* = .539; Zhao 2023 IVW: Q_𝑃 = .499, MR-Egger: Q_*P* = .576), RANKL (IVW: Q_*P* = .418, MR-Egger: Q_*P* = .395), and RANK (IVW: Q_*P* = .279, MR-Egger: Q_*P* =.449). The results indicated no heterogeneity in the analysis of the causal effects between OPG, RANKL, RANK, and scoliosis. The MR-Egger intercept and MR-PRESSO tests for horizontal pleiotropy both yielded *P*-values >.05, indicating that the MR analyses did not exhibit horizontal pleiotropy (Table 2; Leave-one-out plot: Fig. S1A–D, Supplemental Digital Content, http://links.lww.com/MD/O181, Forest plot: Fig. S2A–D, Supplemental Digital Content, http://links.lww.com/MD/O182, Funnel plot: Fig. S3A–D, Supplemental Digital Content, http://links.lww.com/MD/O183). These sensitivity analyses provide some evidence of the reliability and robustness of the findings.

**Table 2:** Results of the test for heterogeneity and horizontal pleiotropy between groups in the MR analysis.

Exposure	Outcome	Pleiotropy test		Heterogeneity test	
		MR-Egger intercept *P* val	MR-Presso Global test_*P* val	IVW Q_*P* val	MR-Egger Q_*P* val
OPG (Folkersen et al)	Scoliosis	.907	.712	.665	.539
OPG (Zhao et al)	Scoliosis	.139	.487	.499	.576
RANKL	Scoliosis	.534	.402	.418	.395
RANK	Scoliosis	.061	.261	.279	.449
Scoliosis	OPG (Folkersen et al)	.740	NA	.637	.398
Scoliosis	OPG (Zhao et al)	.353	NA	.244	.636
Scoliosis	RANKL	.841	NA	.580	.312
Scoliosis	RANK	.492	NA	.405	.384

Abbreviations: NA = not available, OPG = osteoprotectin, Q_*P* val = the *P* val value of Cochran *Q* test, RANK = receptor activator of nuclear factor of kappa-B, RANKL = receptor activator of kappa-B ligand of nuclear factor of kappa-B.

## 7. Causal effect of scoliosis on the RANK-RANKL-OPG system

**Figure 4.**
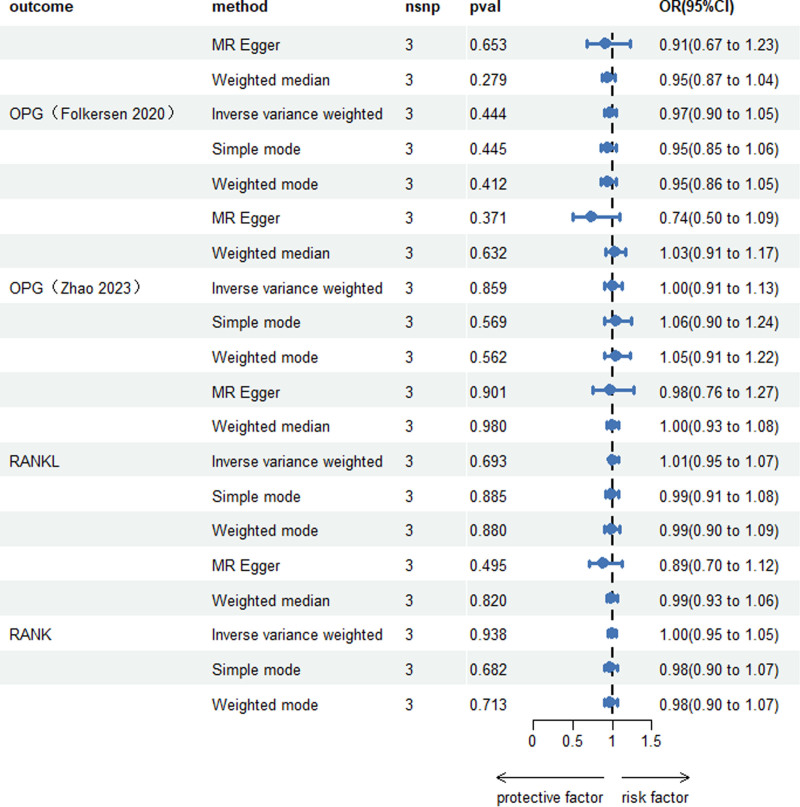
Causal effect of Scoliosis on the RANK-RANKL-OPG system. CI = confidence interval, IVW = inverse variance weighted, NSNP = number of single nucleotide polymorphisms, OPG = osteoprotectin, RANK = receptor activator of nuclear factor of kappa-B, RANKL = receptor activator of kappa-B ligand of nuclear factor of kappa-B.

When scoliosis was exposure, *P* < 5e−8 failed to extract SNPs, so we narrowed the *P*-value to 5e−7 and extracted 3 SNPs (rs1244564, rs140463745, and rs17178928) used as a means of determining the causal effect of scoliosis on OPG, RANKL, and RANK levels, and all SNPs used for MR analysis met the *F*-statistic values > 10. The MR analysis indicated that scoliosis was not associated with OPG, RANKL, or RANK (Fig. 4). Sensitivity analyses showed no signs of heterogeneity or horizontal pleiotropy, as all *P*-values for Cochran *Q* in IVW and MR-Egger, MR-Egger intercept, and MR-PRESSO Global Test exceeded .05 (Table 2). Figs. S1E–H (Supplemental Digital Content, http://links.lww.com/MD/O181), S2E–H (Supplemental Digital Content, http://links.lww.com/MD/O182), and S3E–H (Supplemental Digital Content, http://links.lww.com/MD/O183) display the leave-one-out analysis plots, forest plots, and funnel plots for the causal relationship between scoliosis with RANK-RANKL-OPG.

## 8. Discussion

Previous epidemiological studies have identified a correlation between low bone mass and scoliosis across various populations.^[5]^ The RANK-RANKL-OPG system plays a pivotal role in the process of bone metabolism. RANK, a type I membrane-spanning protein, is found on osteoclast progenitor cells, mature osteoclasts, and cells of the immune system. The RANKL on the osteoblast membrane binds to RANK on the osteoclast progenitor cells, activating NF-kB, JNK, and c-Myc, stimulating osteoclast formation, bone remodeling, and calcium homeostasis.^[26,27]^ OPG, which belongs to the TNF receptor superfamily, is produced by osteoblasts and functions as a decoy receptor for RANKL. It competitively inhibits the differentiation and function of osteoclasts. The ratio of RANK/OPG indicates bone health, reflecting the balance between bone formation and resorption.^[28]^

MR is a genetic method for estimating causal relationships between traits. Genetic studies are minimized by environmental factors, which is an advantage of MR over epidemiological studies. In the present study, we performed a bidirectional 2-sample MR analysis using a large genomic research database to assess the causal relationship between the RANK-RANKL-OPG system and scoliosis. Meta-analysis was used to compile the results of the IVW method to further improve reliability. The results showed that OPG has a protective effect against scoliosis, with low levels of OPG potentially increasing the risk of scoliosis. This finding is consistent with the known role of OPG in inhibiting osteoclastogenesis and maintaining bone balance. Eun found that the 1181G -> C polymorphism in the OPG gene is linked to lumbar BMD in female patients with adolescent idiopathic scoliosis, which aligns with our findings.^[29]^

The possible mechanism by which OPG influences the risk of scoliosis is that reduced OPG levels lead to increased RANKL-RANK expression, exacerbating osteoclast-mediated bone resorption, resulting in decreased bone mineral density (BMD) and impaired bone quality. This imbalance in bone remodeling can cause asymmetric spinal growth, making vertebrae more susceptible to deformation under mechanical loading. BMD is considered a factor in predicting curve progression in scoliosis patients, and low BMD during critical growth periods may lead to the development of scoliosis. An MR analysis showed a causal relationship between BMD and the risk of AIS in European females, with low BMD increasing the risk of AIS.^[30]^ In adulthood, lower bone density and impaired bone quality make vertebrae more susceptible to deformation under mechanical loading, leading to degenerative scoliosis. Additionally, OPG contributes to bone metabolism and exerts significant effects on immune response, vascular protection, and other physiological processes.^[31]^ Animal studies have shown that mice lacking OPG display muscle frailty and selective muscle shrinkage.^[32]^ Bonnet demonstrated that inhibiting RANKL and supplementing OPG can enhance muscle robustness and recuperate bone density in mice with osteoporosis.^[33]^ We hypothesize that low levels of OPG may increase the risk of scoliosis by affecting bone mass. However, the study did not find a causal relationship between RANKL and RANK with scoliosis. The mechanism by which OPG affects scoliosis is unclear and requires further research. Lower OPG levels may be an important predictive factor for future screening and therapy of scoliosis.

Nevertheless, this study presents certain limitations. First, the GWAS data collection included only populations of European ancestry. Although this helps reduce potential bias from population stratification and genetic heterogeneity within the study cohort, it restricts the broader applicability of the MR analysis outcomes to populations with diverse genetic compositions. Second, the analysis data were not stratified by sex and age, which may lead to biased results. The levels of RANKL and OPG are affected by factors such as age and gender. RANKL expression tends to rise with age, whereas OPG expression stays fairly stable. This could result in a higher RANKL/OPG ratio as 1 age, which is linked with enhanced bone resorption and an elevated risk of osteoporosis.^[34]^ A study suggested that the sex-determining region Y (SRY), a male-specific transcription factor, reduces RANKL expression. In healthy males, RANKL expression surpasses that in healthy females by 20%, suggesting that variations in RANKL levels and bone mass between sexes could stem from the sex-specific transcription factor SRY.^[35]^ Additionally, OPG levels are generally higher in females than in males, and menopause affects OPG levels in women.^[36,37]^

In conclusion, this study used a bidirectional 2-sample MR analysis to explore the causal relationship between the RANK/RANKL/OPG system with scoliosis. The findings confirm a causal link between diminished OPG levels and heightened susceptibility to scoliosis, providing new insights into the pathophysiology of scoliosis. These results underscore the role of OPG as a potential biomarker for early diagnosis and a target for therapeutic intervention in scoliosis, laying the foundation for further research into targeted treatments for scoliosis. However, the complexity of OPG and its interactions with host genetics and environmental factors require further research to elucidate these associations fully. Future studies should consider potential confounding factors, such as diet and lifestyle, that may influence OPG and RANKL levels.

## 9. Conclusion

Our study using MR methodology yields robust evidence supporting the causal relationship between decreased OPG levels and increased susceptibility to scoliosis. However, there was no significant relationship between scoliosis with the RANKL/RANK/OPG system. It lays the foundation for further exploration of the pathophysiological mechanisms and potential targeted treatment of scoliosis. Future research is necessary to fully comprehend how OPG affects scoliosis.

## Acknowledgments

We are very grateful to the participants and investigators of the UK Biobank, the FinnGen study and the European Institute for Bioinformatics for making our study possible.

## Author contributions

**Conceptualization:** Wei Xie, Wen-Tao Wan.

**Data curation:** Wei Xie, Shuai-yi Liu, Jia-Qi Wang.

**Formal analysis:** Wei Xie.

**Funding acquisition:** Qiang Yang.

**Methodology:** Qiang Yang.

**Project administration:** Qiang Yang.

**Resources:** Xin-Yu Liu.

**Software:** Jia-Qi Wang.

**Supervision:** Wen-Tao Wan, Chao Chen, Xun Sun, Xin-Yu Liu, Qiang Yang.

**Writing – original draft:** Wei Xie.

**Writing – review & editing:** Wen-Tao Wan, Shuai-Yi Liu, Chao Chen, Xun Sun, Xin-Yu Liu, Qiang Yang.

## Supplementary Material


