# Supplementary figures and images for: Causal effects of the RANK-RANKL-OPG system and scoliosis: A bidirectional 2-sample Mendelian randomization study
Source: Medicine (Baltimore). 2024 Dec 13;103(50):e40934. doi: 10.1097/MD.0000000000040934 (PMC11651458; doi:10.1097/MD.0000000000040934)

Figure 1 A

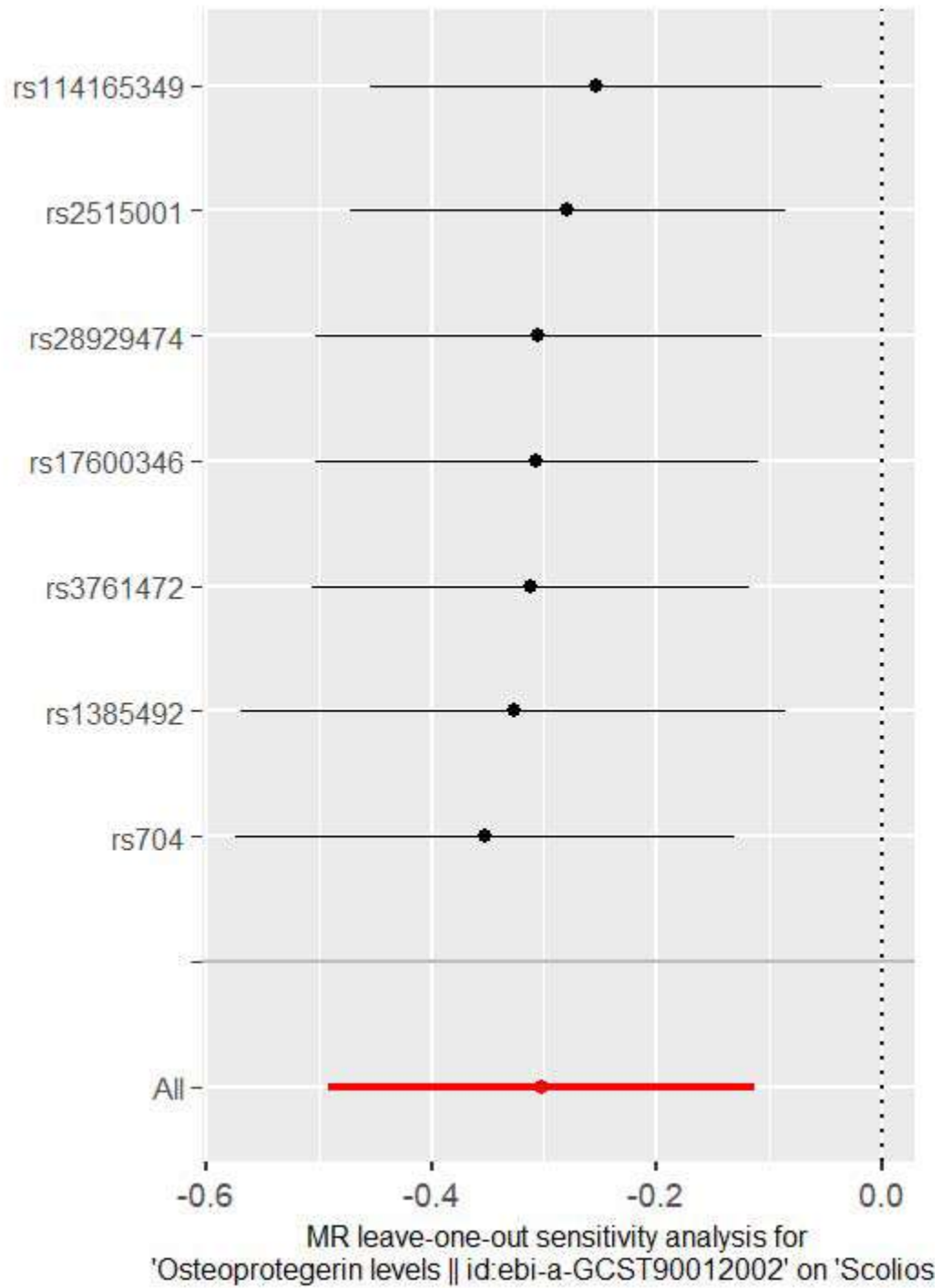

Figure 1 B

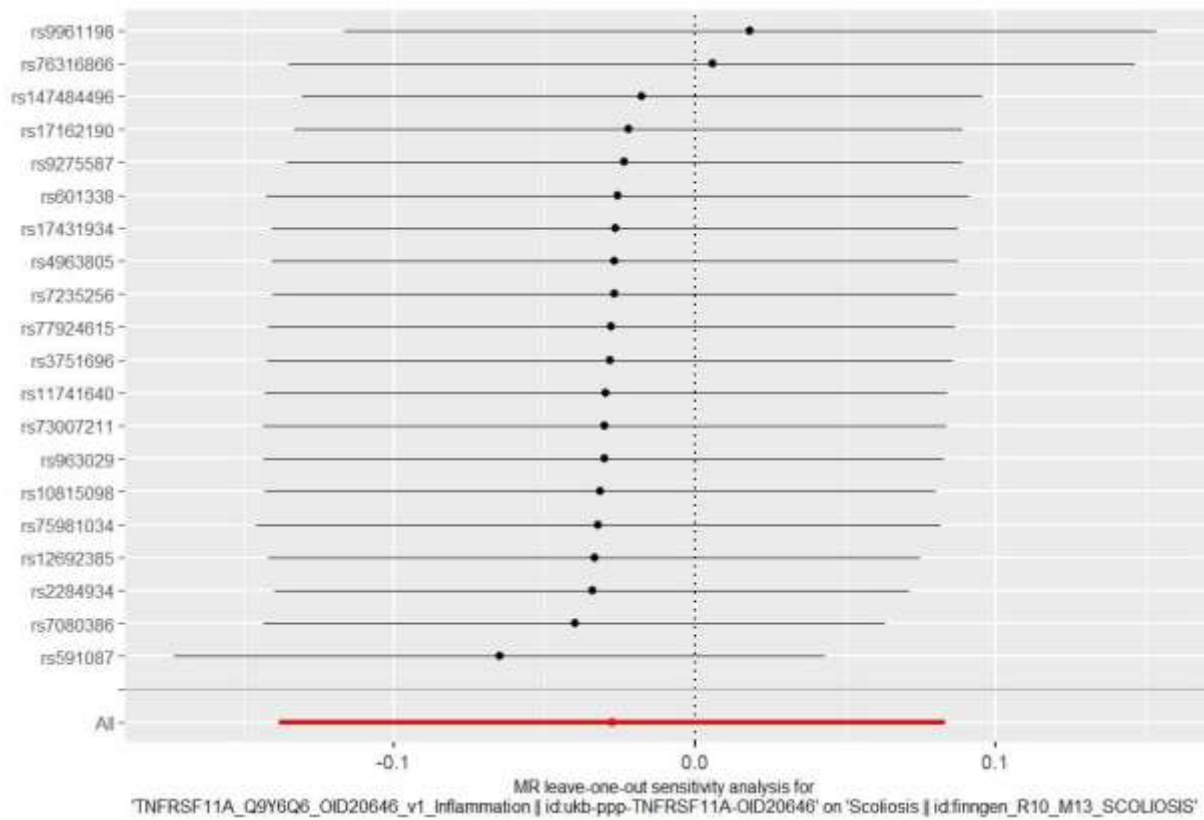

Figure 1 C

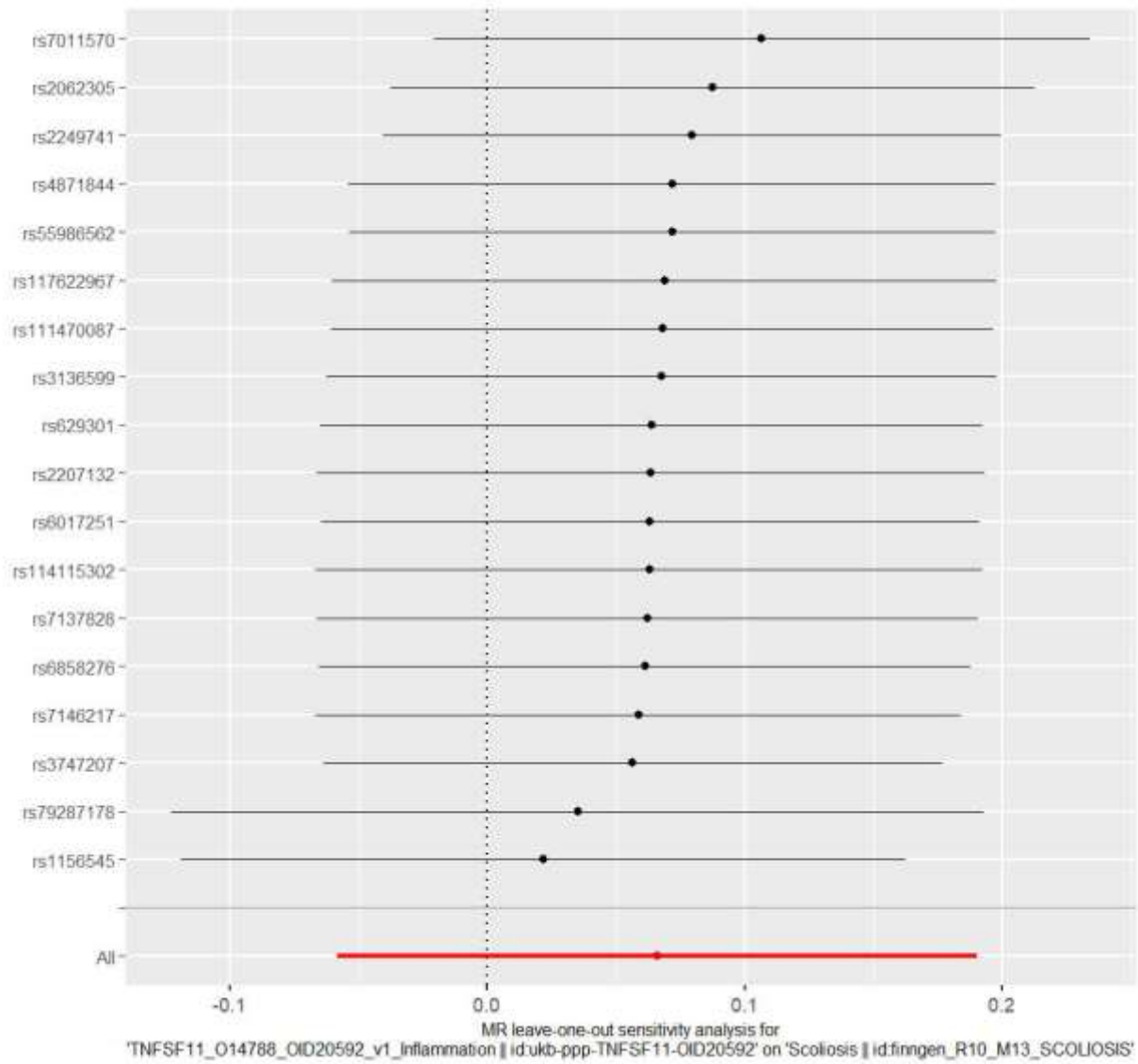

Figure 1 D

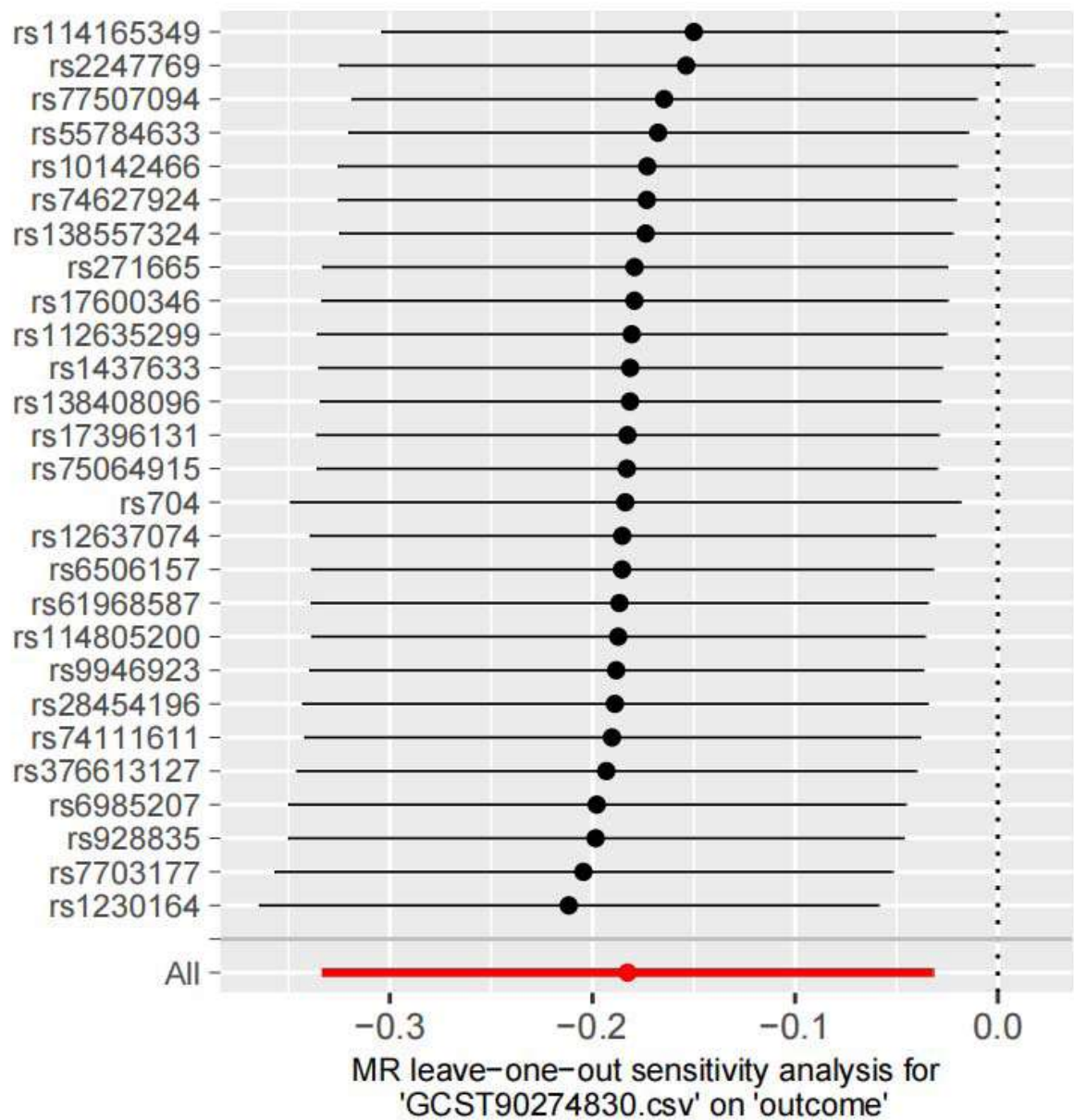

Figure 1 E

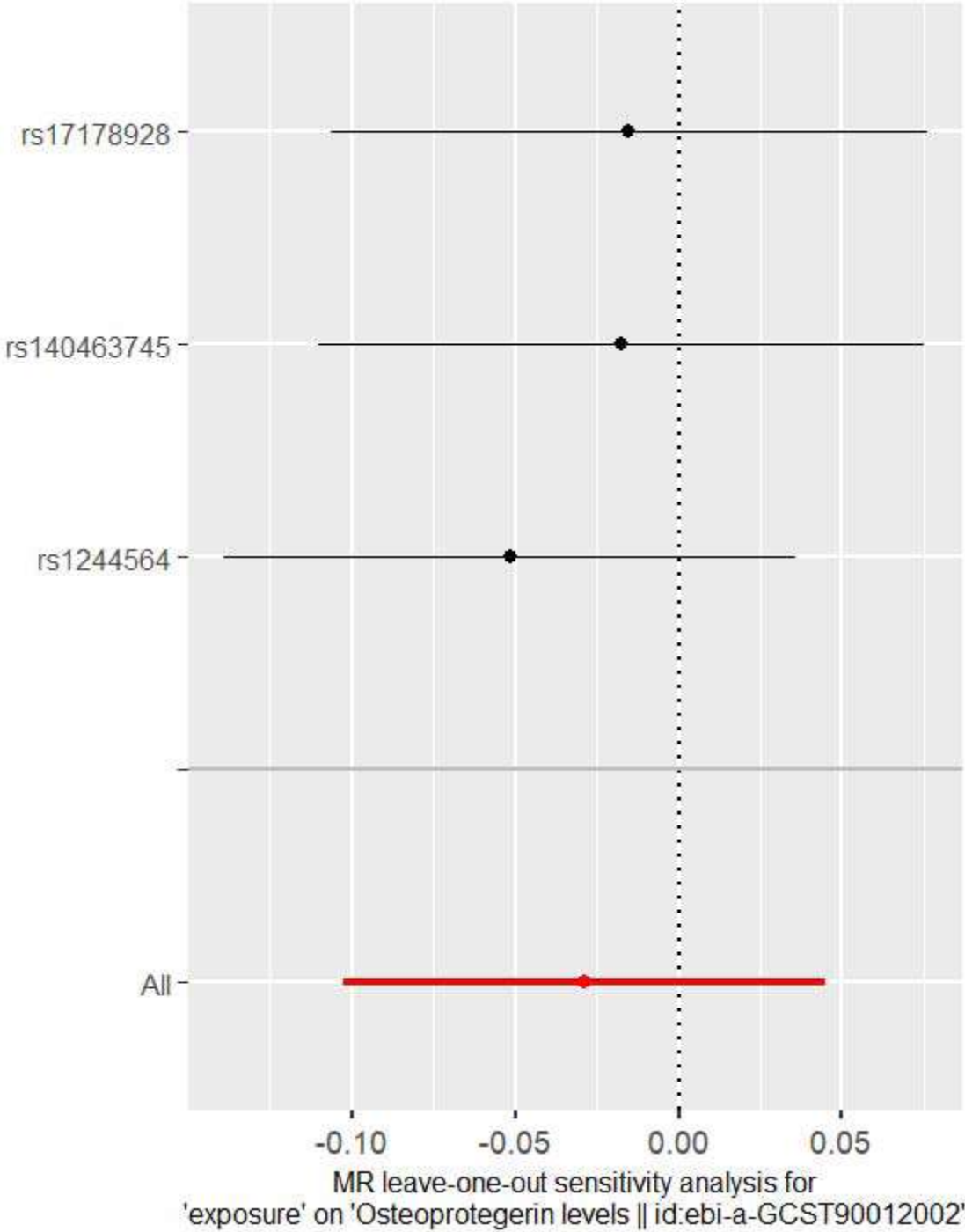

Figure 1 F

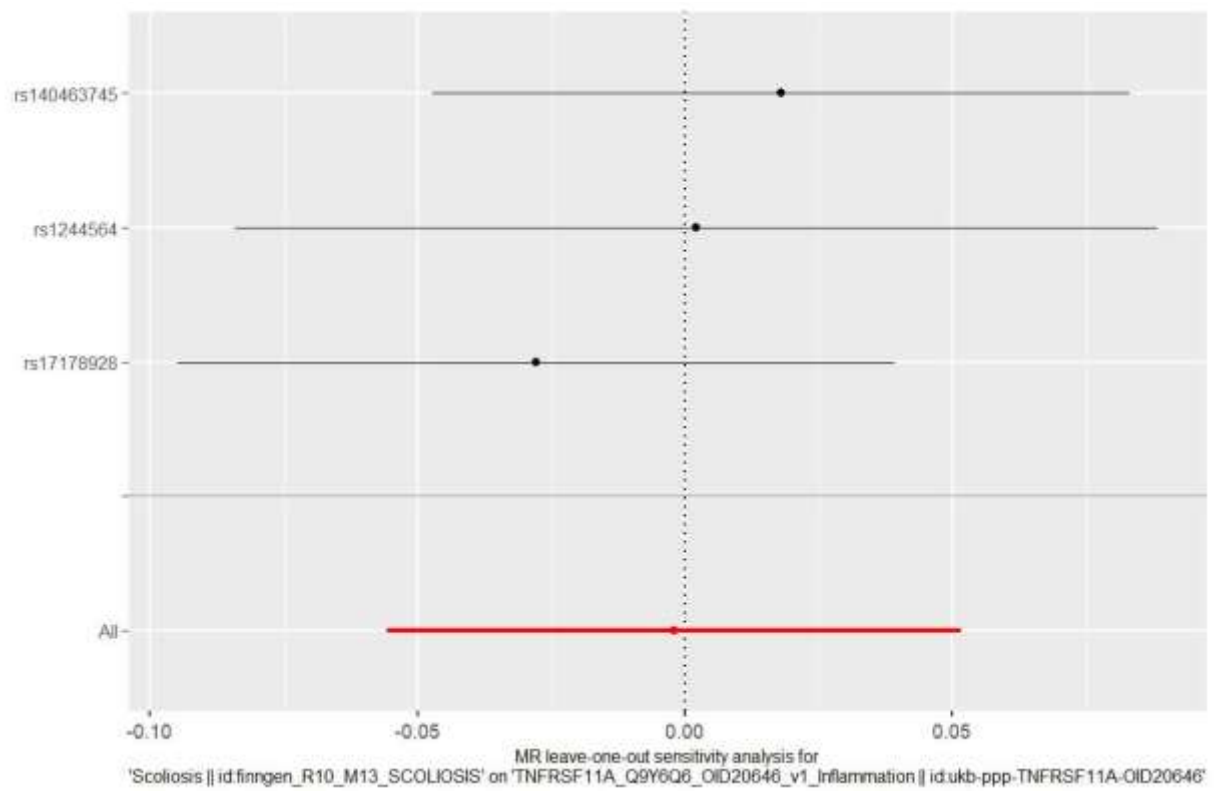

Figure 1 G

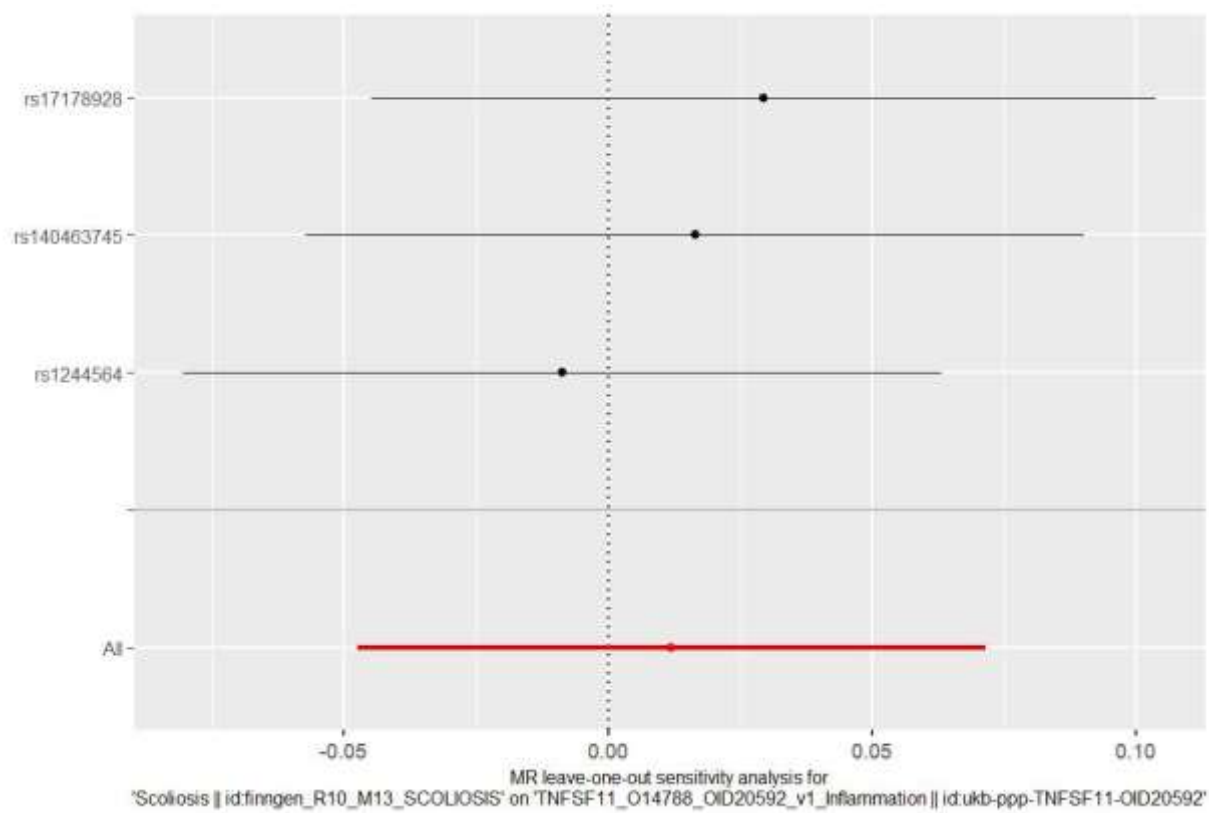

Figure 1 H

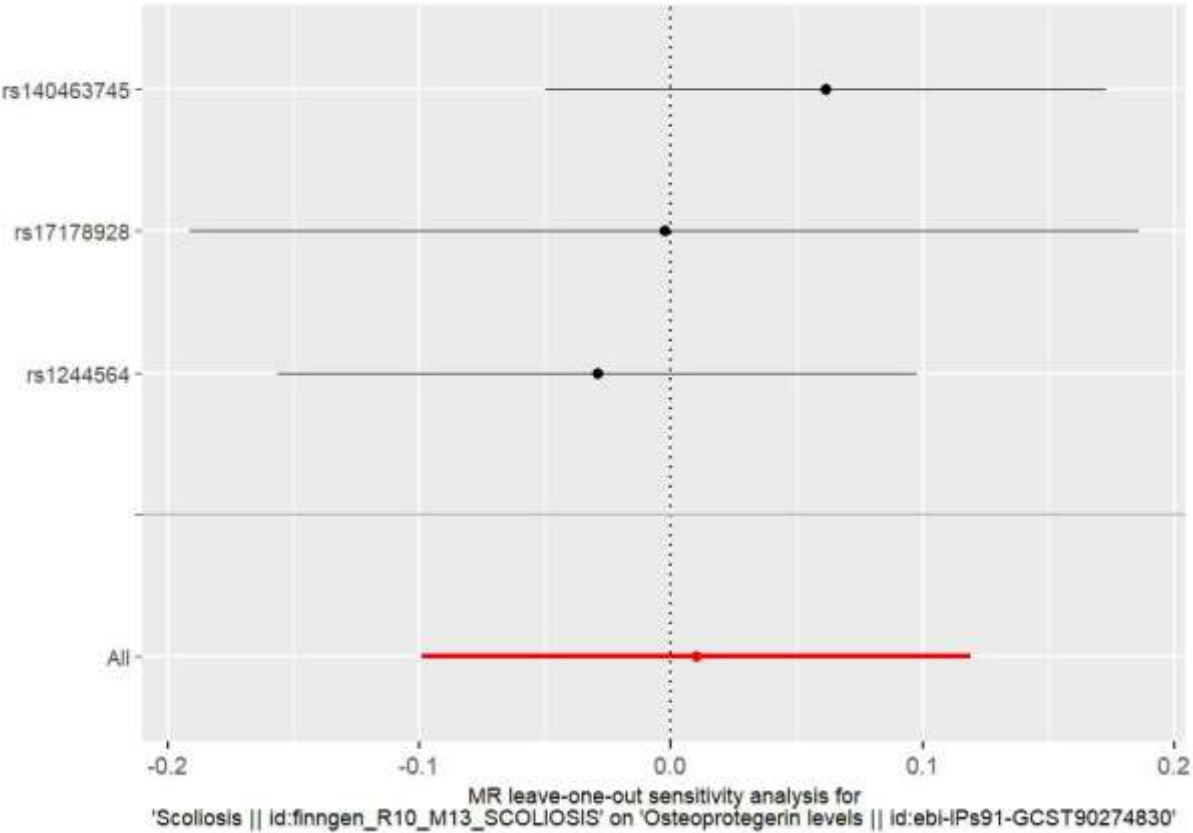

Supplement: Supplementary file 1 [file medi-103-e40934-s001.pdf]

Figure 2 A

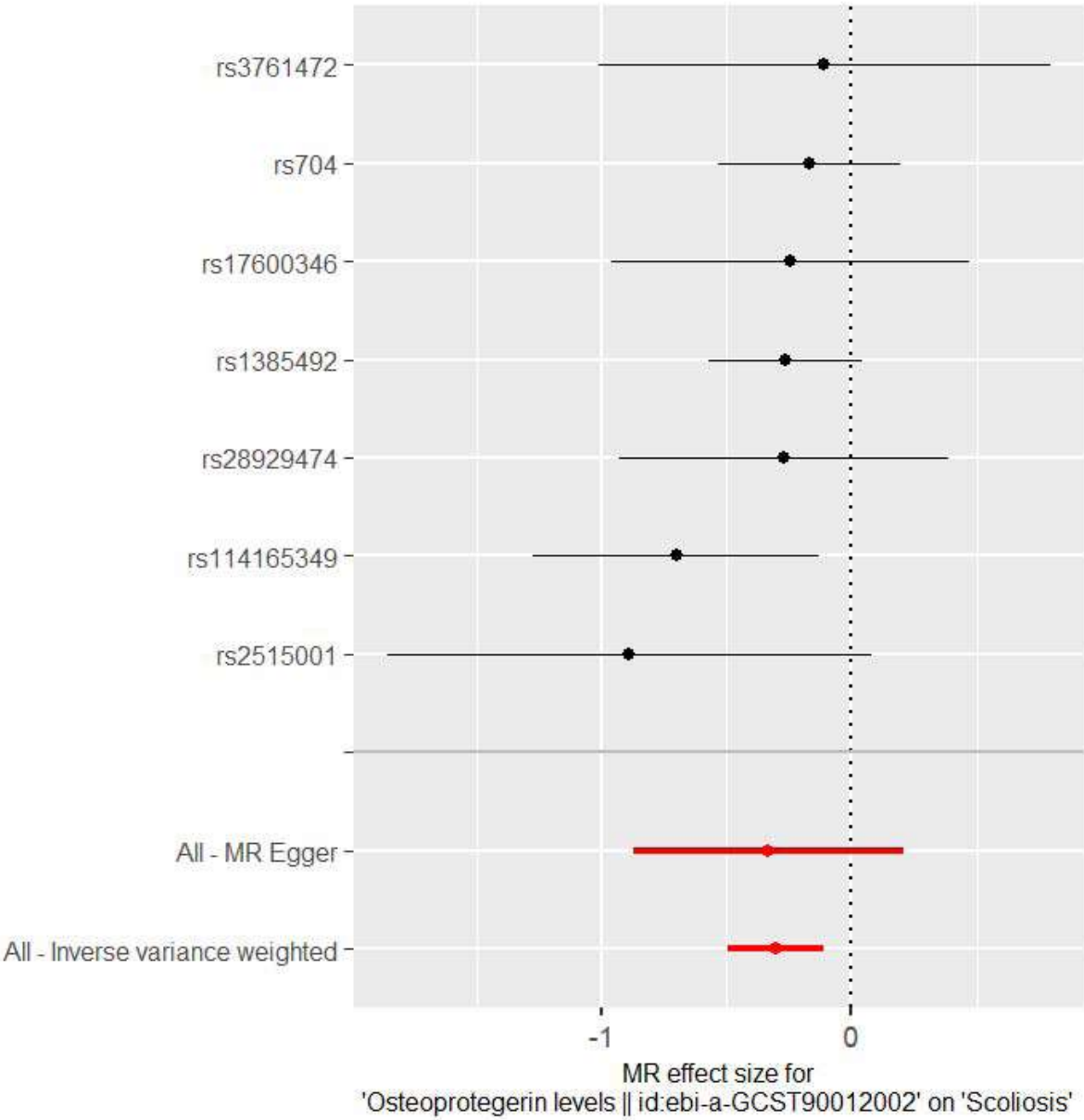

Figure 2 B

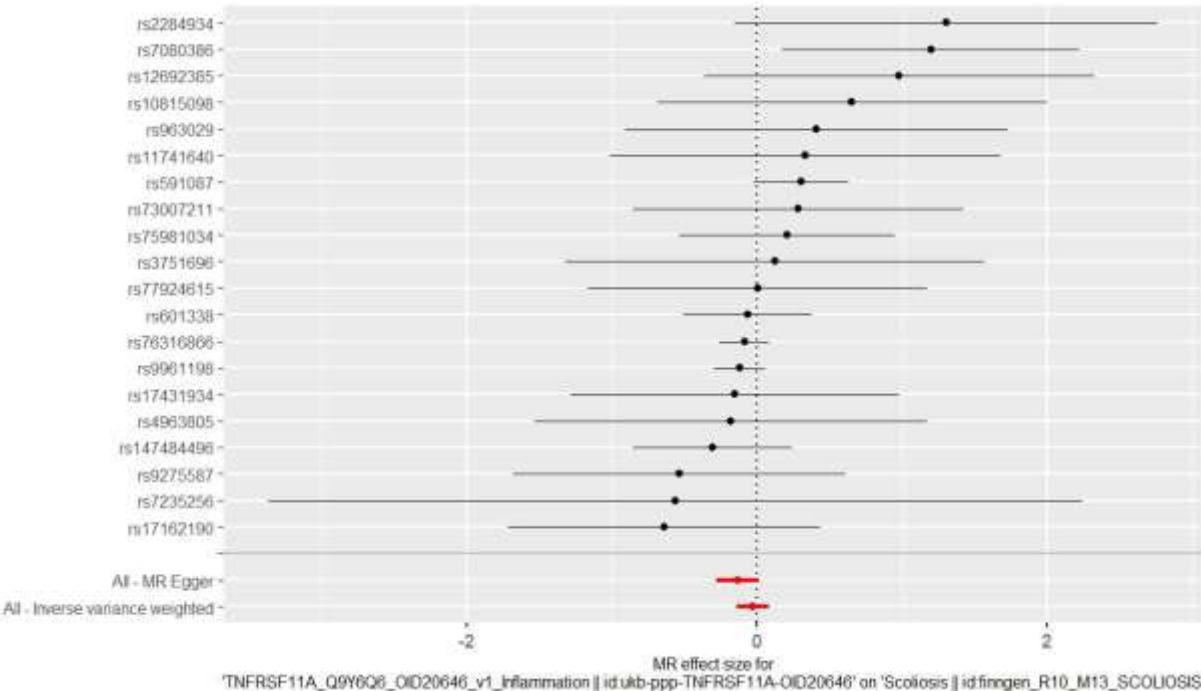

Figure 2 C

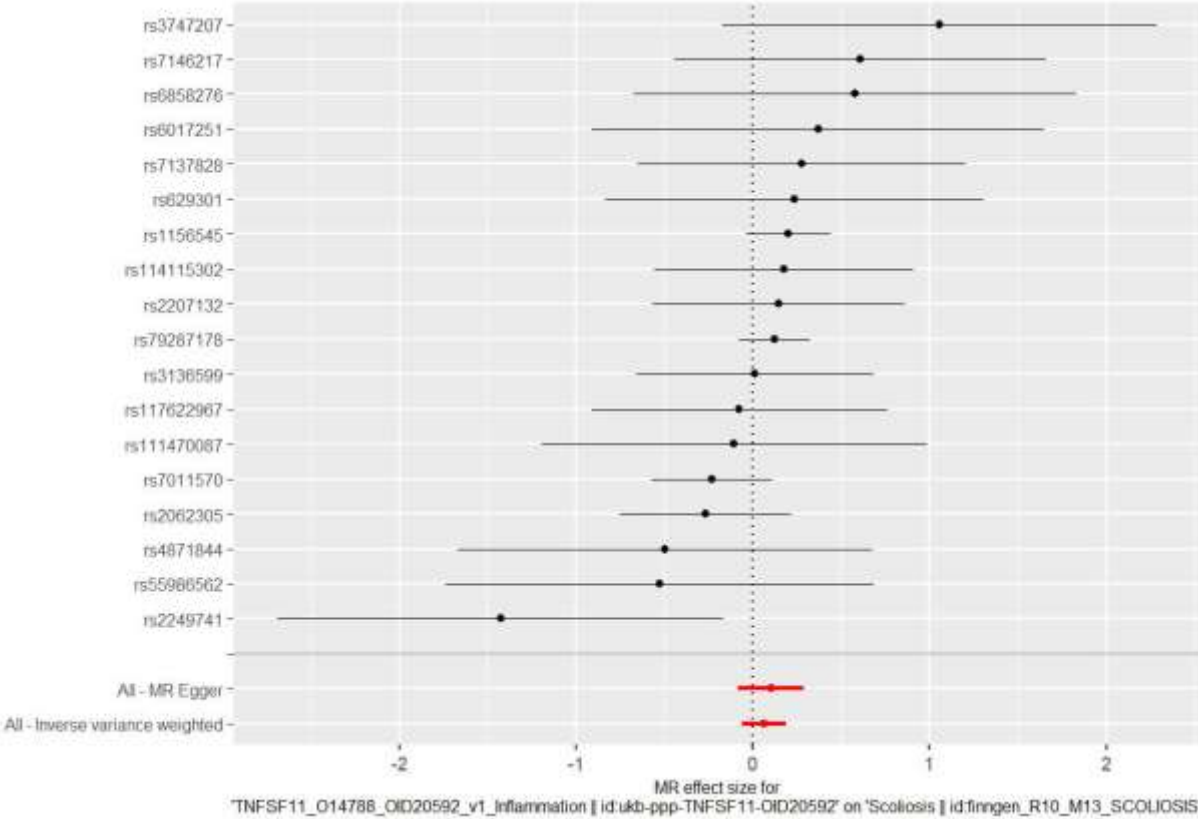

Figure 2 D

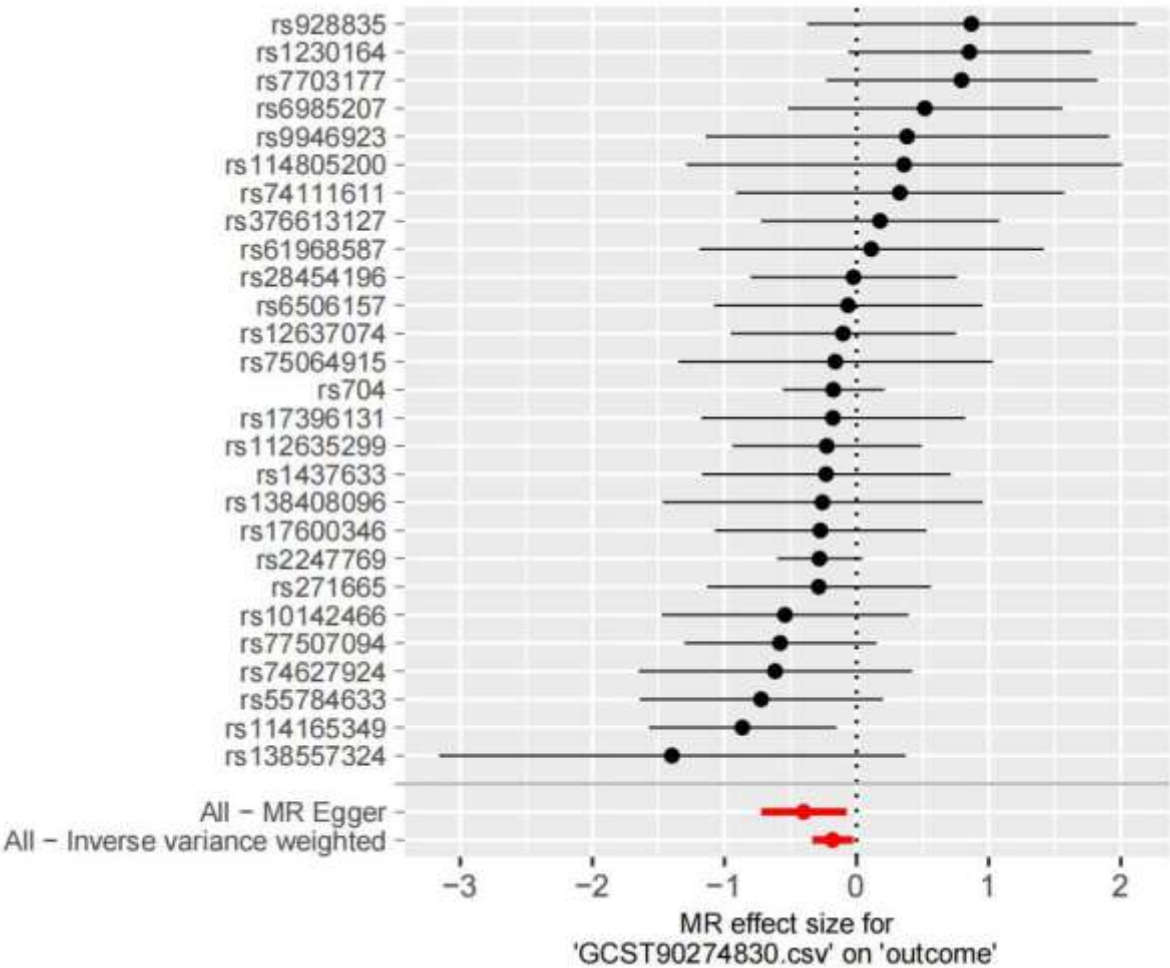

Figure 2 E

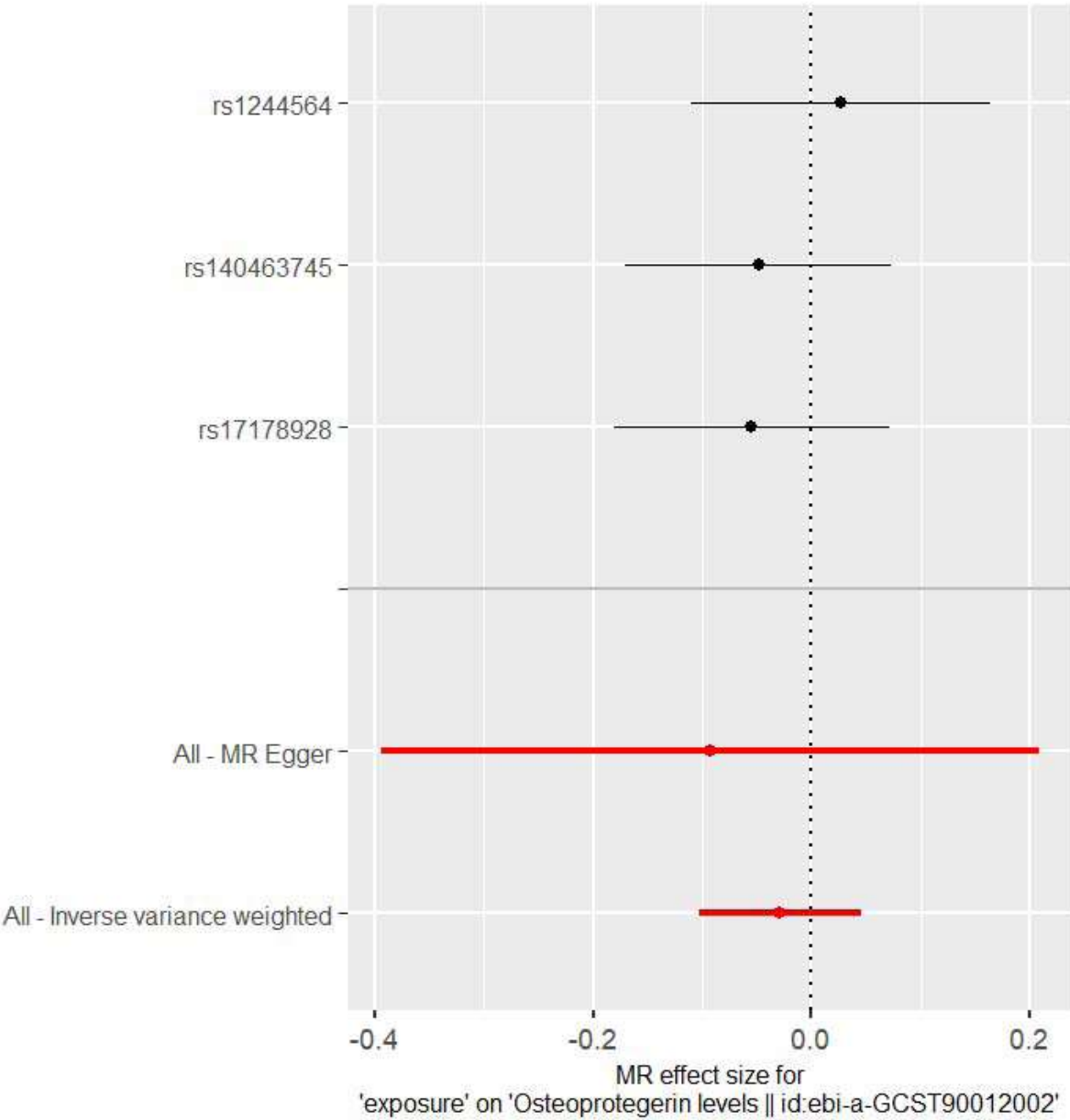

Figure 2 F

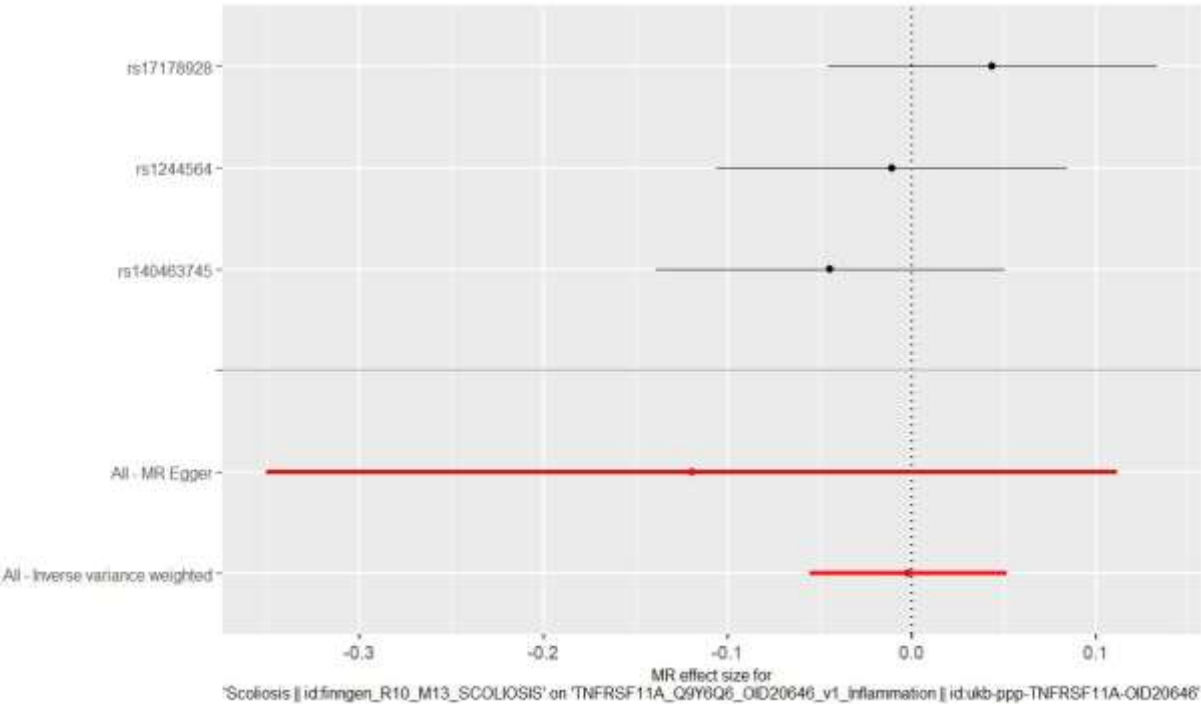

Figure 2 G

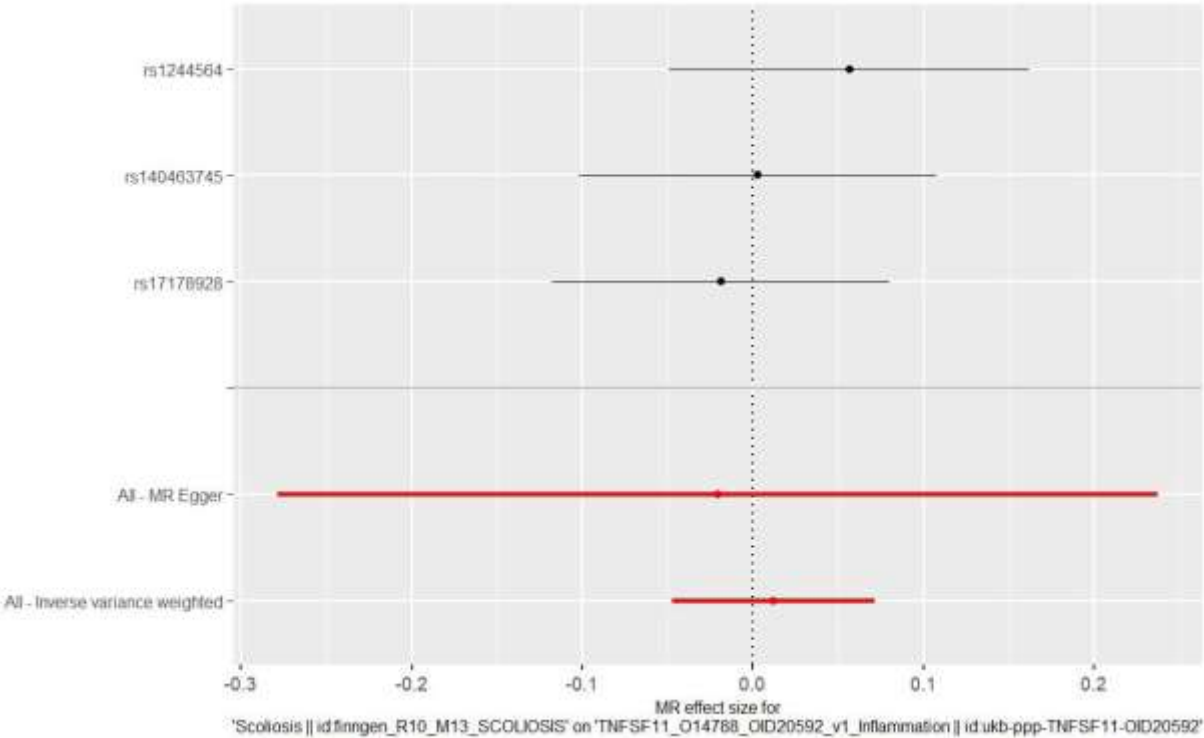

Figure 2 H

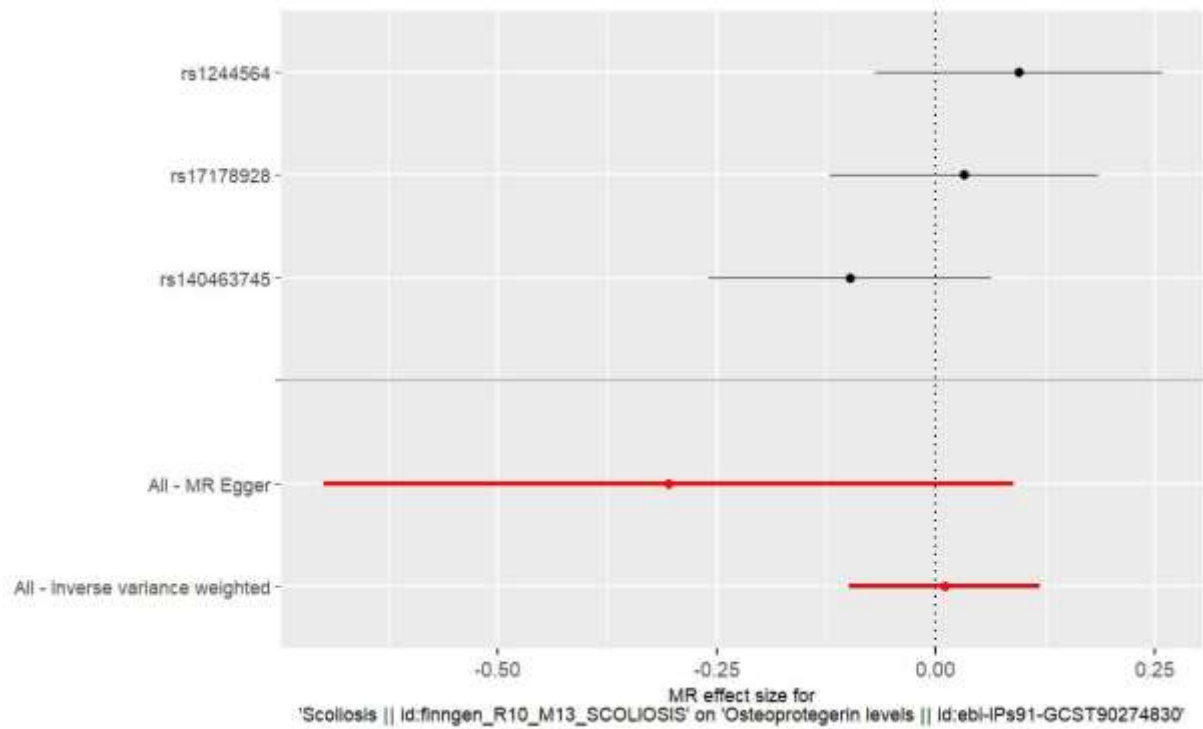

Supplement: Supplementary file 2 [file medi-103-e40934-s002.pdf]

Figure 3 A

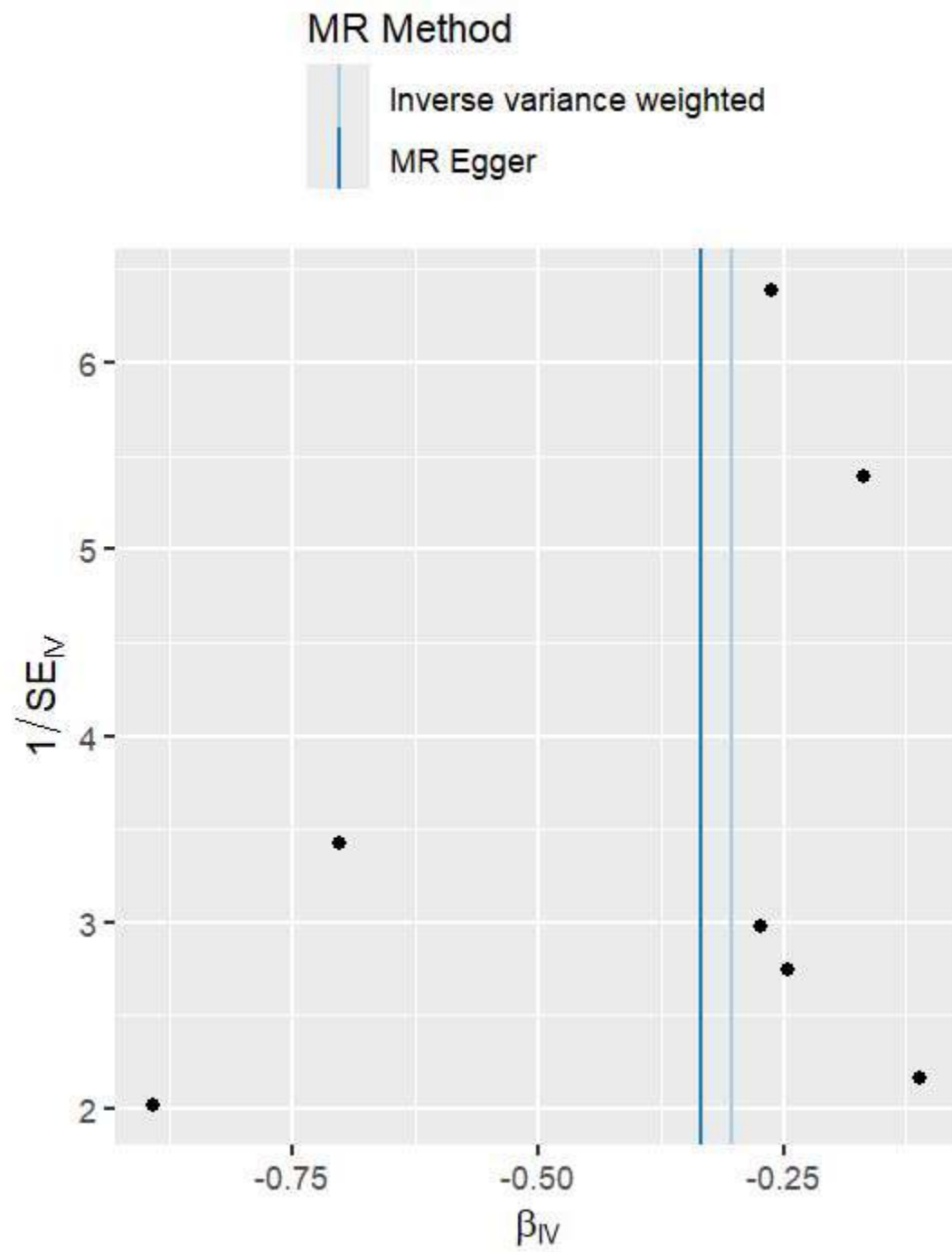

Figure 3 B

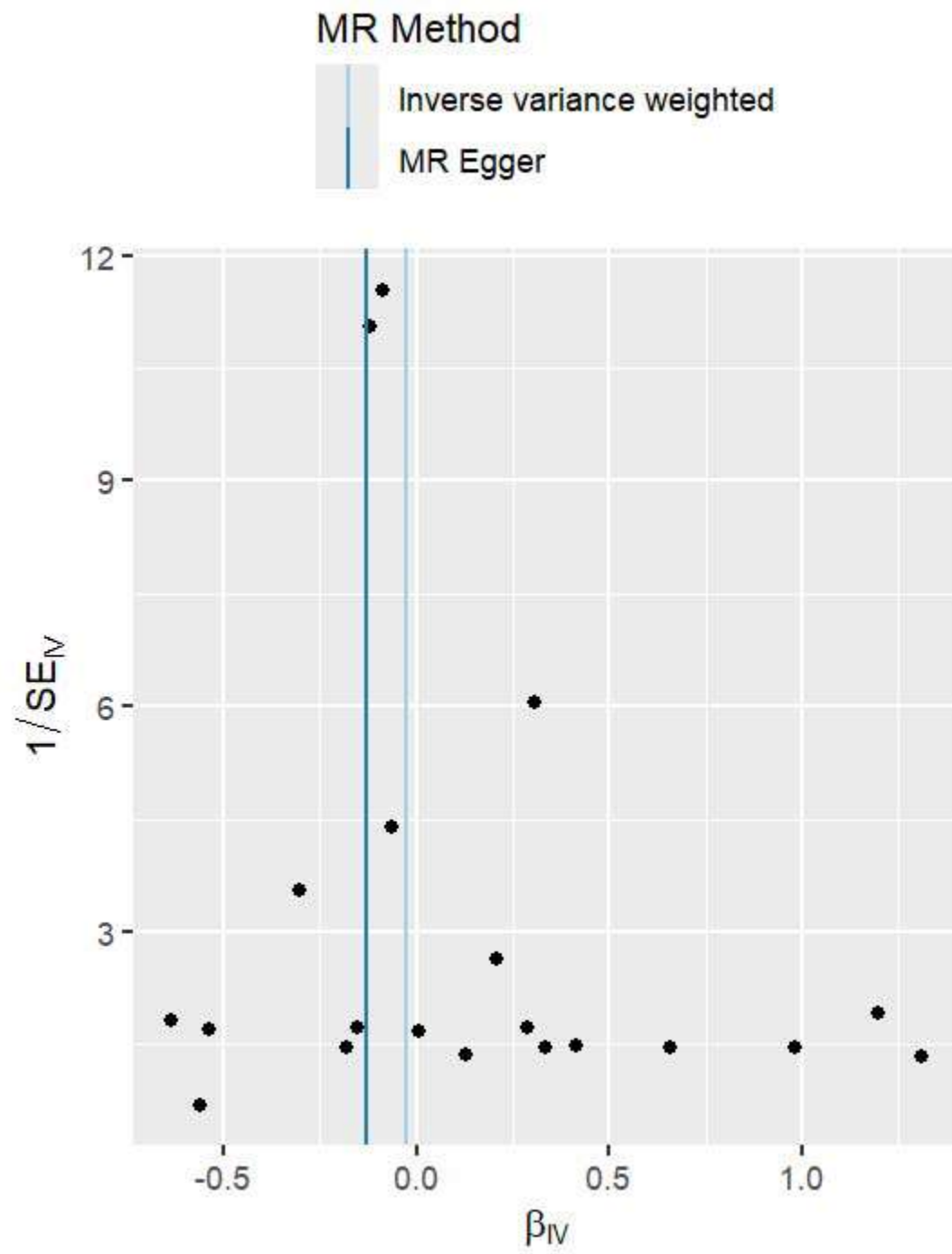

Figure 3 C

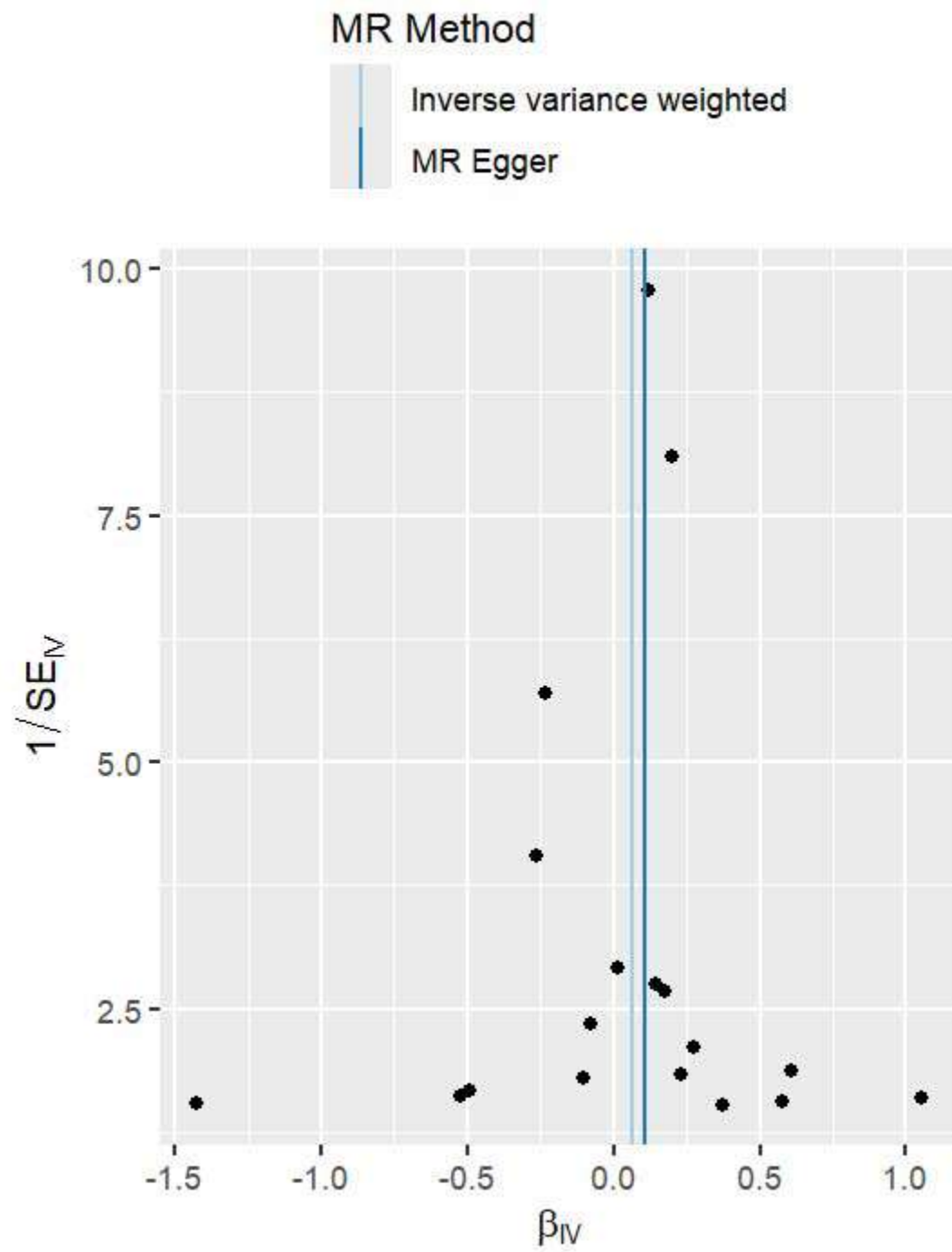

Figure 3 D

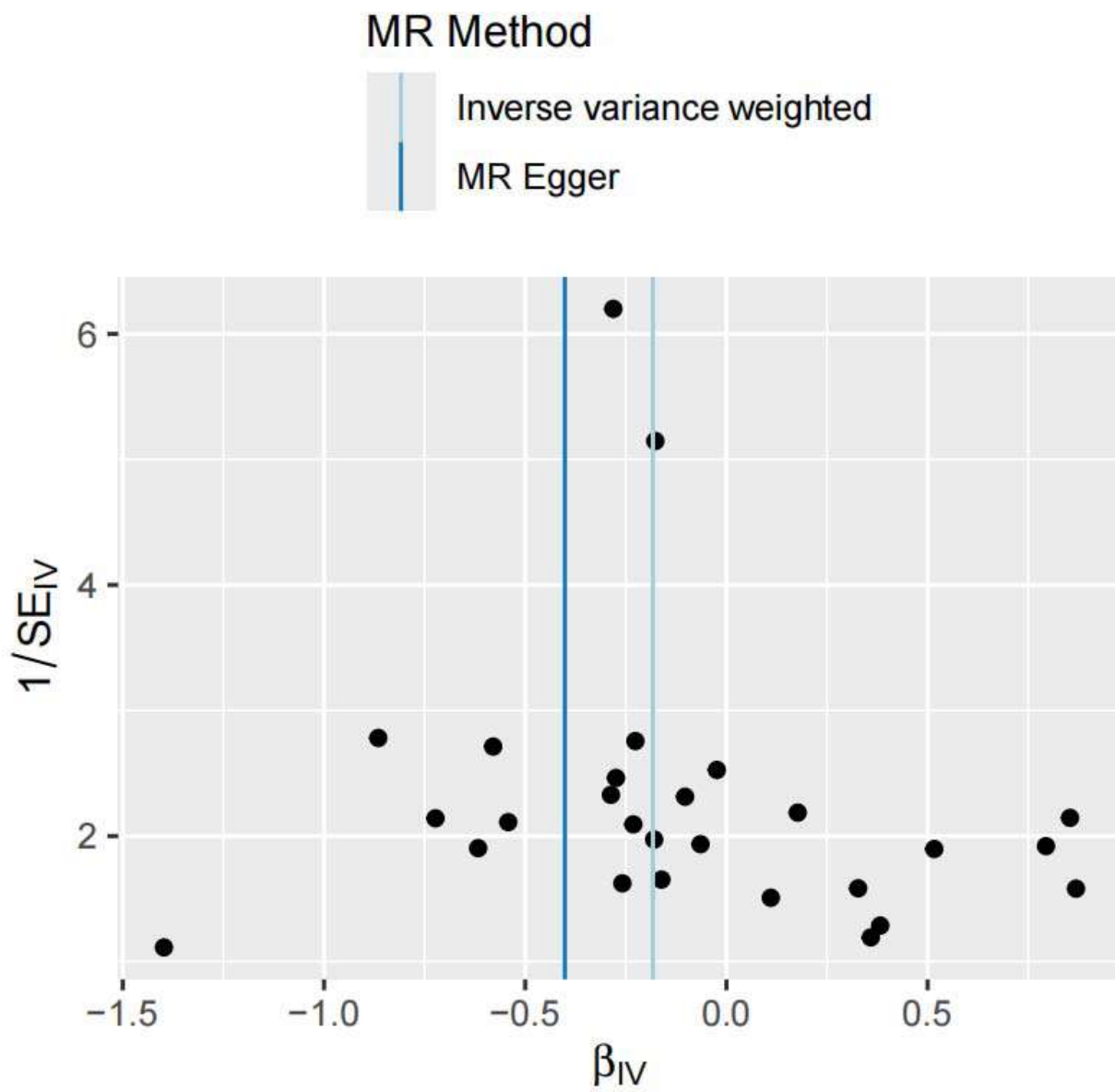

Figure 3 F

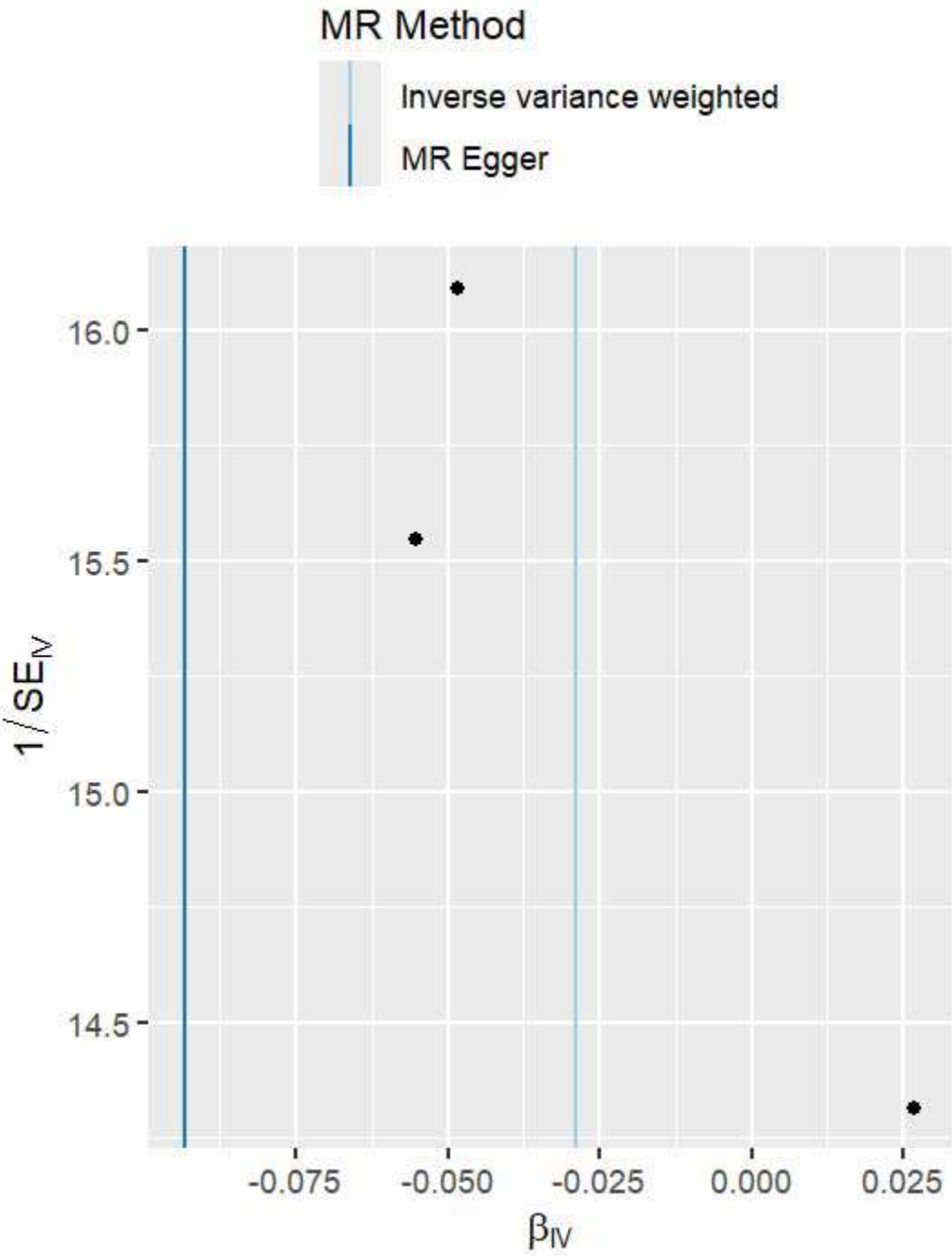

Figure 3 F

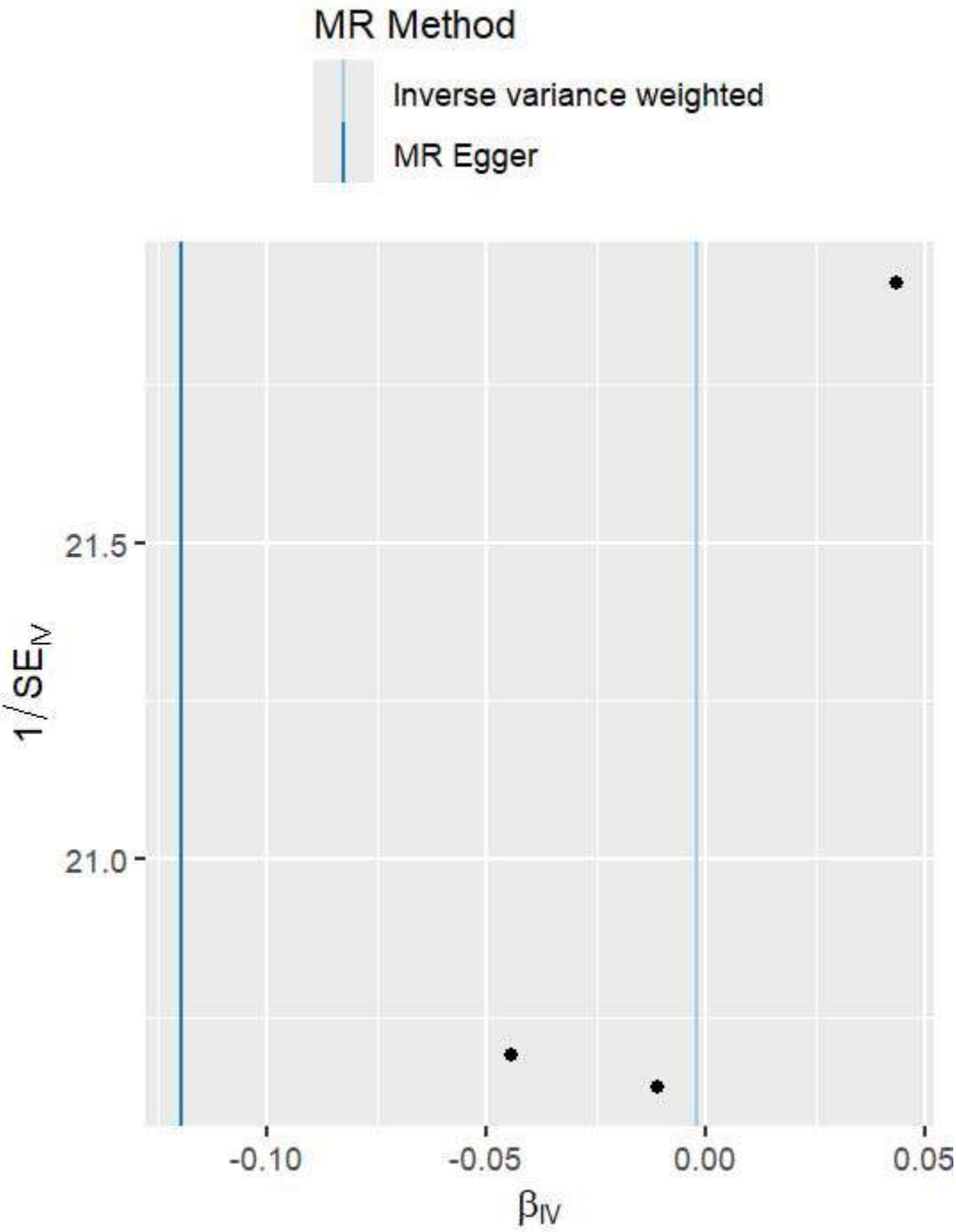

Figure 3 G

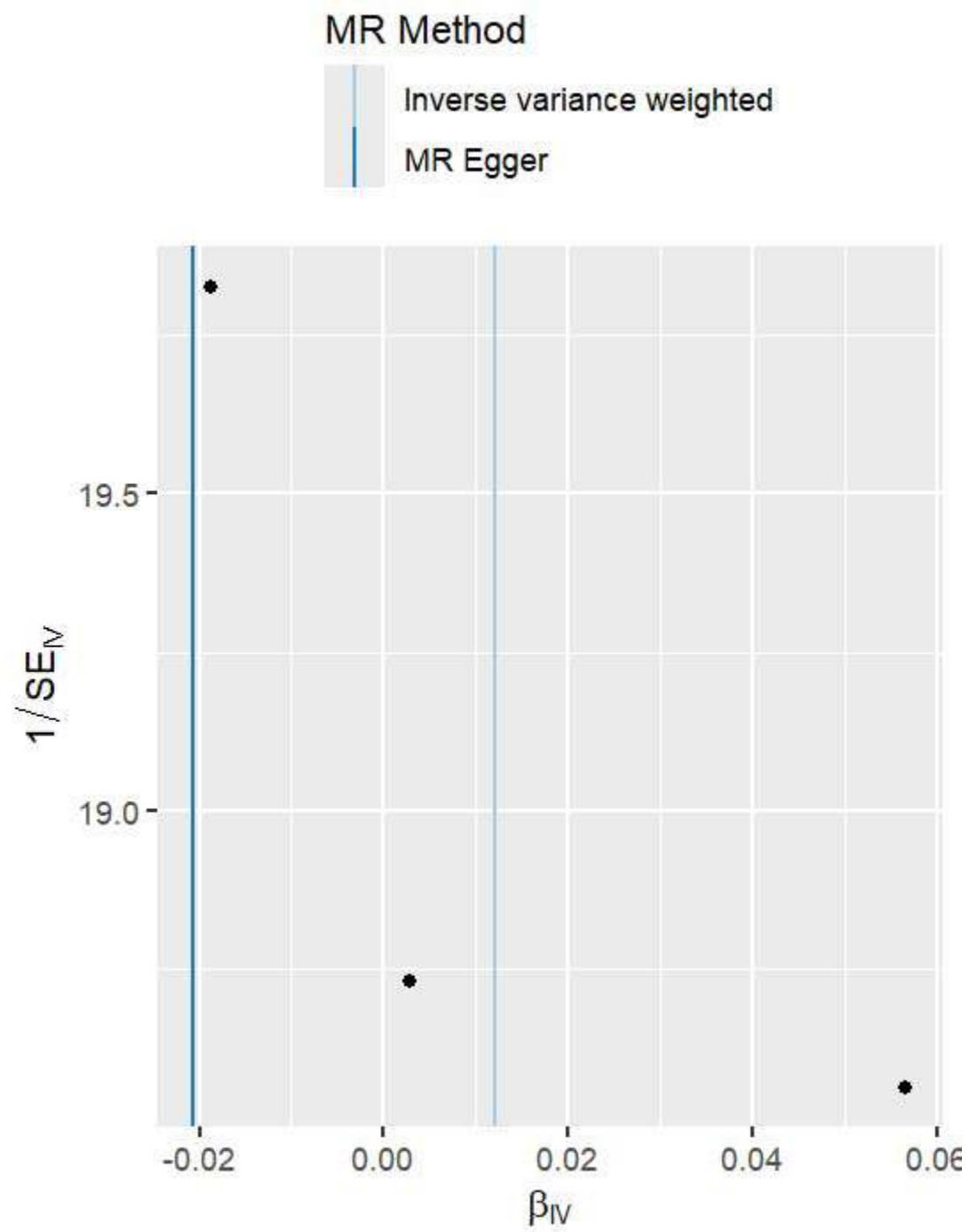

Figure 3 H

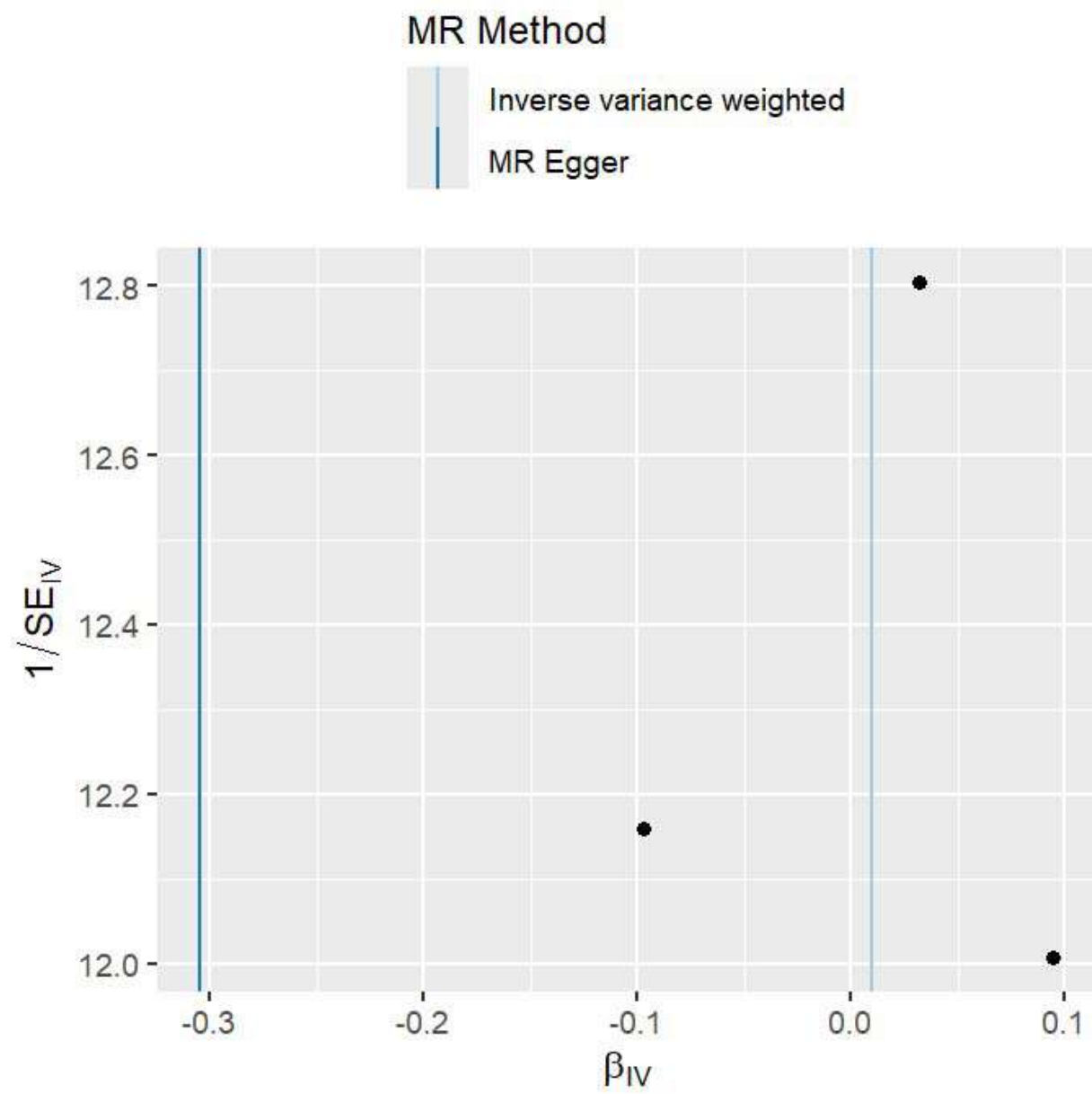

Supplement: Supplementary file 3 [file medi-103-e40934-s003.pdf]
